# Cognitive behavioral therapy for irritable bowel syndrome induces bidirectional alterations in the brain-gut-microbiome axis associated with gastrointestinal symptom improvement

**DOI:** 10.1186/s40168-021-01188-6

**Published:** 2021-11-30

**Authors:** Jonathan P. Jacobs, Arpana Gupta, Ravi R. Bhatt, Jacob Brawer, Kan Gao, Kirsten Tillisch, Venu Lagishetty, Rebecca Firth, Gregory D. Gudleski, Benjamin M. Ellingson, Jennifer S. Labus, Bruce D. Naliboff, Jeffrey M. Lackner, Emeran A. Mayer

**Affiliations:** 1 G. Oppenheimer Center for Neurobiology of Stress and Resilience, UCLA, Los Angeles, CA USA; 2 David Geffen School of Medicine, UCLA, Los Angeles, CA USA; 3 Vatche and Tamar Manoukian Division of Digestive Diseases, UCLA, Los Angeles, CA USA; 4 Division of Gastroenterology, Hepatology and Parenteral Nutrition, Veterans Administration Greater Los Angeles Healthcare System, Los Angeles, CA USA; 5 Imaging Genetics Center, Mark and Mary Stevens Institute for Neuroimaging and Informatics, Keck School of Medicine at USC, University of Southern California, Los Angeles, USA; 6 Division of Behavioral Medicine, Jacobs School of Medicine, University at Buffalo, SUNY, Buffalo, NY USA; 7 Department of Radiological Sciences, UCLA, Los Angeles, CA USA; 8 Department of Psychiatry and Biobehavioral Sciences, UCLA, Los Angeles, CA USA; 9 G. Oppenheimer Center for Neurobiology of Stress and Resilience, Vatche and Tamar Manoukian Division of Digestive Diseases, David School of Medicine at UCLA, CHS 42-210 MC737818, 10833 Le Conte Avenue, Los Angeles, USA

**Keywords:** Cognitive behavioral therapy, Irritable bowel syndrome, Brain-gut-microbiome axis, Neuroimaging, Biomarkers, Outcome prediction

## Abstract

**Background:**

There is growing recognition that bidirectional signaling between the digestive tract and the brain contributes to irritable bowel syndrome (IBS). We recently showed in a large randomized controlled trial that cognitive behavioral therapy (CBT) reduces IBS symptom severity. This study investigated whether baseline brain and gut microbiome parameters predict CBT response and whether response is associated with changes in the brain-gut-microbiome (BGM) axis.

**Methods:**

Eighty-four Rome III-diagnosed IBS patients receiving CBT were drawn from the Irritable Bowel Syndrome Outcome Study (IBSOS; ClinicalTrials.gov NCT00738920) for multimodal brain imaging and psychological assessments at baseline and after study completion. Fecal samples were collected at baseline and post-treatment from 34 CBT recipients for 16S rRNA gene sequencing, untargeted metabolomics, and measurement of short-chain fatty acids. Clinical measures, brain functional connectivity and microstructure, and microbiome features associated with CBT response were identified by multivariate linear and negative binomial models.

**Results:**

At baseline, CBT responders had increased fecal serotonin levels, and increased Clostridiales and decreased *Bacteroides* compared to non-responders. A random forests classifier containing 11 microbial genera predicted CBT response with high accuracy (AUROC 0.96). Following treatment, CBT responders demonstrated reduced functional connectivity in regions of the sensorimotor, brainstem, salience, and default mode networks and changes in white matter in the basal ganglia and other structures. Brain changes correlated with microbiome shifts including *Bacteroides* expansion in responders.

**Conclusions:**

Pre-treatment intestinal microbiota and serotonin levels were associated with CBT response, suggesting that peripheral signals from the microbiota can modulate central processes affected by CBT that generate abdominal symptoms in IBS. CBT response is characterized by co-correlated shifts in brain networks and gut microbiome that may reflect top-down effects of the brain on the microbiome during CBT.

Video abstract

**Supplementary Information:**

The online version contains supplementary material available at 10.1186/s40168-021-01188-6.

## Introduction

Irritable bowel syndrome (IBS) is a common disorder of brain-gut interactions [1, 2] defined by recurrent abdominal pain associated with altered bowel habits in the absence of any gastrointestinal structural, inflammatory, immunologic, or biochemical pathology [3]. Due to the lack of reliable biomarkers capable of IBS classification, symptom improvement and efficacy of treatment are usually assessed by patient-reported measures [4, 5].

The growing recognition of the important role of brain-gut-microbiome (BGM) interactions in regulating GI function, symptom perception, mood, and affect has marked it as a target for therapeutic intervention in IBS [6, 7]. A body of evidence supports the presence of anatomical and functional connectivity alterations in brain networks in IBS patients related to emotional arousal, salience assessment, sensorimotor, and brainstem function (reviewed in [8]). In addition, several reports have identified alterations in the relative abundances of microbial taxa in subsets of patients [9, 10], including a study demonstrating an association of specific gut microbial alterations with differences in grey matter volumes of sensory- and salience-related regions [11].

Cognitive behavioral therapy (CBT) is an effective brain-targeted intervention that teaches information processing skills to address psychological factors known to exacerbate abdominal symptoms including maladaptive coping, intense worry (e.g., catastrophizing, prediction error), stress reactivity, and hypervigilance to threat cues [12, 13]. We have recently shown in a large randomized clinical trial that two CBT programs tailored for IBS were effective in producing sustained gastrointestinal symptom improvement compared to an IBS education program that controlled for nonspecific effects from undergoing treatment [14]. To the extent that CBT induces symptomatic change through biological pathways, we hypothesized that this occurs by modulating primarily the brain component of the BGM axis, but the effect of these central changes on the rest of the BGM axis and on symptom improvement are not known. We further hypothesized that microbial signals to the brain in the form of neuroactive metabolites including short-chain fatty acids and serotonin could modulate responsiveness to the biological effects of CBT [15].

We prospectively recruited 84 IBS participants from the parent randomized, controlled, parallel CBT trial (the Irritable Bowel Syndrome Outcome Study) [14] and assessed brain resting-state functional connectivity and microstructure at baseline and 2 weeks following CBT treatment. Composition and function of the gut-microbiome were characterized in 34 CBT recipients. By assessing relative abundances and functions of the gut microbiota, multimodal brain imaging, and detailed clinical measures, we aimed to test two major hypotheses: (1) Do baseline brain and/or microbiome parameters predict treatment outcomes? (2) Is successful treatment response to CBT associated with alteration of brain and microbiome parameters?

## Methods

### Study oversight

Institutional Review Boards at each of two sites approved the protocols governing participants: University at Buffalo (12/18/2012-12/13/2017) and Northwestern University (11/21/2012-10/21/2015). An independent Data Safety Monitoring Board appointed by the National Institute of Diabetes and Digestive and Kidney Diseases monitored the study bi-annually for participant safety, study conduct, and progress. Patients and the public were involved in the development and qualitative testing of clinical materials used in this study.

### Participants

IBS participants recruited for this study of biological mechanisms related to IBS symptom improvement after CBT came from the larger parent study, the IBSOS (*n*=436), the results of which have been published previously [14]. Detailed inclusion and exclusion criteria have been previously published [14, 16]. In brief, adults aged between 18 and 70 years diagnosed with IBS via Rome III criteria [17] who rated their symptoms as “moderately severe” (i.e., occurred at least twice weekly and caused life interference) were included. Patients were excluded if they had another primary GI disease, malignancy in the past 5 years, major psychiatric comorbidity, were undergoing IBS-targeted psychotherapy, could not commit to completing all scheduled visits, reported a gastrointestinal infection within 2 weeks before evaluation, or used a gut-sensitive antibiotic during the 12 weeks prior to evaluation. Eighty-four participants underwent neuroimaging and detailed clinical assessment, and thirty-four participants underwent assessment of microbiome parameters and dietary intake (Diet History Questionnaire II; further description provided in the Supplementary Methods). These participant numbers were based on funding availability, and no selection criteria were applied beyond the inclusion/exclusion criteria of the parent study.

### Trial procedures

Eligible patients were randomized to receive 10 sessions of clinic-based CBT or 4 sessions of largely home-based CBT with minimal therapist contact over a 10-week acute phase as previously described [14]. The 10 session version was delivered once a week for 10 weeks, while the 4 session version was delivered at week 1, week 3, week 5, and week 10. For these analyses, the two CBT protocols were combined as they include the same components and procedures (patient education, self-monitoring, muscle relaxation, worry control, flexible problem solving, relapse prevention) and have been shown to have efficacy equivalence [14, 18].

### Clinical measures

Clinical measures were obtained at pre-treatment baseline and 2 weeks after completion of CBT. The 2 week delay was intended to reduce the effects of treatment related processes such as the relationship with the therapist and expectation for improvement. Details of the measures used in the larger study have been previously published [18, 19]. The IBS Symptom Severity Scale (IBS-SSS) [4] was used to measure symptom severity including pain, distention, bowel dysfunction, and quality of life/global well-being. The threshold for clinical improvement was set a priori at a decrease of 50 points or greater [4]. Participants meeting this criterion were classified as a responder. Additional clinical measures included IBS self-efficacy, pain unpleasantness, perceived stress, and mood (further description provided in the Supplementary Methods).

### Magnetic resonance imaging

All participants underwent baseline and post-treatment imaging sessions in a 3T Siemens Prisma MRI Scanner (Siemens, Erlangen, Germany) for a high-resolution T1 structural scan, resting-state functional scan, and diffusion tensor image (details of acquisition and processing provided in the Supplementary Methods).

### Statistical analysis of neuroimaging data

Baseline differences and pre-post CBT treatment changes in resting-state functional connectivity were measured separately in responders and in non-responders. A paired *t* test controlling for age and sex, using the general linear model (GLM) framework in CONN, was conducted on all connections of the average 165 × 165 matrix before and after CBT. This was done separately for CBT responders and then for CBT non-responders. Significance was set at *α*=0.05, and all tests were corrected using the Benjamini-Hochberg procedure at the seed-level. Visualizations were done using circos 0.69 in Linux [20].

The voxelwise associations between baseline and pre-post treatments changes in diffusion tensor imaging (DTI) parameters were evaluated separately in responders and non-responders to CBT within white matter and subcortical structures including the basal ganglia, thalamus, and brainstem. Statistical parametric maps (SPM) were generated using a GLM that accounted for both age and sex. The model involved comparing SPMs that quantified the difference in change in fractional anisotropy (FA) or apparent diffusion coefficient (ADC) as a result of treatment in CBT responders and then for non-responders. The GLM was implemented using AFNI (https://afni.nimh.nih.gov). The resulting SPMs were thresholded at a level of significance *p*<0.05 for each contrast of interest, and a cluster size threshold greater than 250 μL. This minimum cluster size is similar to previous studies using cluster-based permutation correction [21–23], while also trying to maximize the sensitivity to DTI changes.

### Fecal 16S rRNA gene sequencing

Fresh frozen fecal samples were obtained from 34 patients in this cohort after the baseline screening visit and within 2 weeks after the end of treatment. Participants were provided with home stool collection kits and were asked to store their stool samples in a freezer immediately after collection until pick up by a courier service within 24–48 h of collection for storage at −80°C. Frozen fecal samples were later ground into a coarse powder by mortar and pestle under liquid nitrogen then aliquoted. DNA extraction by bead beating and amplification of the V4 hypervariable region of the 16S rRNA gene were performed according to our published protocol [24, 25]. The library underwent 2×250 sequencing on an Illumina HiSeq 2500 to a mean depth of 107,433 merged sequences per sample. QIIME 1.9.1 was used to perform quality filtering, merge paired end reads, and cluster sequences into 97% operational taxonomic units [26]. Taxonomy was assigned using the Greengenes May 2013 database. Microbial alpha diversity was assessed on datasets rarefied to equal sequencing depth (37,662) using the Chao1 index of richness, Faith’s phylogenetic diversity, and the Shannon index of evenness. Microbial composition was compared across samples by weighted UniFrac distances and visualized with principal coordinates analysis [27]. The significance of differences in microbial composition was assessed using multivariate Adonis with 100,000 permutations [28]. Differential abundance of microbial genera at baseline was determined using multivariate negative binomial mixed models implemented in DESeq2 with bowel habit subtype and sex as covariates [29]. Longitudinal Adonis and DESeq2 models comparing pre- vs. post-treatment values included participant identifier as a covariate.

### Fecal metabolomics

Fecal aliquots were shipped to Metabolon, Inc. and run as a single batch on their global HD4 metabolomics platform. Compounds were identified by comparison of spectral features to Metabolon’s proprietary library that includes MS/MS spectral data on more than 3300 purified standards. Results were provided as scaled, imputed abundances of 872 known compounds. Stool aliquots additionally underwent a targeted LC-MS/MS analysis to measure concentrations of nine short-chain fatty acids. Global metabolomics profiles were compared across participants and visualized in two dimensions using the square root of the Jensen-Shannon divergence and non-metric multidimensional scalin g[25]. Stress is a measure used to assess goodness of fit of the reduced dimensions; 0.2 is typically used as a cutoff for suitable fit. Significance of differences between CBT responders and non-responders was determined by Adonis, adjusting for bowel habit and sex. Statistical significance of short-chain fatty acids, serotonin, dopamine, and histamine between CBT responders and non-responders at baseline or between pre- vs. post-CBT values was assessed by the non-parametric Mann–Whitney *U* test. Differential abundance testing across the entire dataset was performed on log-transformed data using GLMs that included bowel habit and sex as covariates. Significance was calculated using empirical Bayes moderated *t* statistics implemented in the limma R package [30]. Participant identifier was utilized as a covariate in Adonis and limma analyses of longitudinal data.

### Random forests classifiers

To determine how well baseline microbiome, clinical, or brain data predicted CBT response (responder/non-responder status), random forests classification with 5-fold cross-validation was implemented using the caret R packag e[31]. The accuracy of the resulting classifiers was determined by calculating the area under the receiver operating characteristic curve (AUROC). Contribution of each variable to classifier accuracy was assessed by variable importance scores, which represent the decrease in classifier accuracy when that variable is permuted. Significance of differences in accuracy of the derived classifiers was assessed using the bootstrap method of roc.test in the pROC R package.

### Correlations between microbiome changes with brain changes and clinical changes

Partial correlations controlling for age and sex were run between significant brain changes and microbiome or clinical changes in CBT responders compared to non-responders. *P* values were adjusted for multiple hypothesis testing with the Benjamini-Hochberg false discovery rate procedure and significant *q* values were reported.

## Results

Of the 84 IBS participants who underwent neuroimaging, 58 (69%) were classified as CBT responders while 26 (31%) were classified as non-responders based on 50-point or greater decrease on the IBS Symptom Severity Scale post-treatment. Baseline clinical and brain-gut-microbiome parameters were compared between CBT responders and non-responders to determine whether treatment outcome could be predicted.

### CBT responders have distinct microbiome profiles at baseline

#### Clinical measures

There were no differences in any of the clinical measures at pre-treatment baseline between responders and non-responders among the 84 subjects who underwent neuroimaging (Table S1).

#### Brain functional connectivity

Responders showed greater baseline connectivity than non-responders between the central autonomic network (right suborbital sulcus) and the emotional regulation network (right inferior frontal sulcus, *q*=.02; right triangular part of the inferior frontal sulcus, *q*=.02).

#### White matter integrity

No statistically significant differences in baseline fractional anisotropy (FA) or apparent diffusion coefficient (ADC) were observed between CBT responders compared to non-responders after adjusting for multiple hypothesis testing.

#### Microbiome composition

**Fig. 1:**
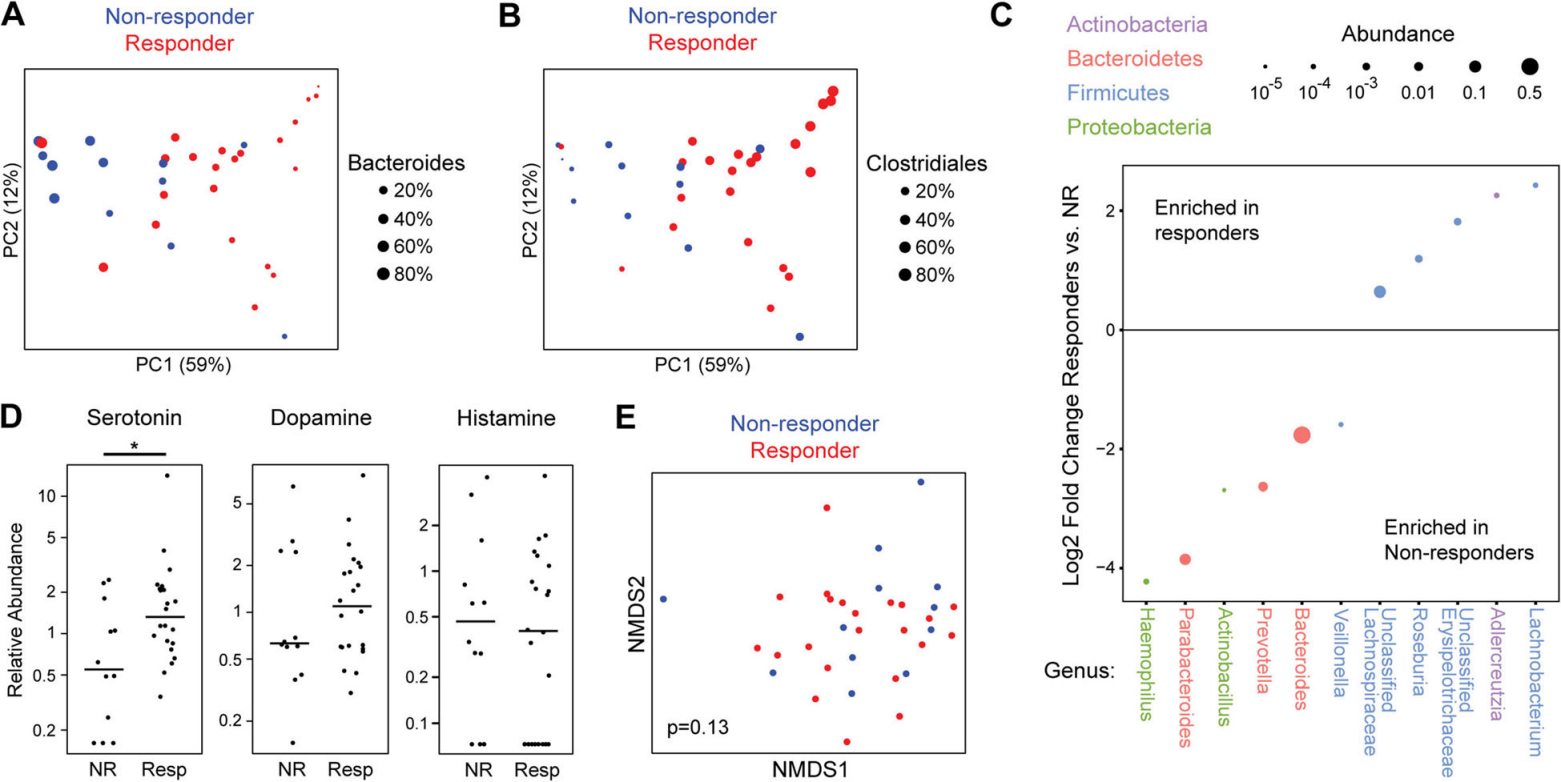
Baseline fecal microbiota and serotonin are associated with CBT response. **A**, **B** Principal coordinates (PC) analysis of 16S rRNA sequence data. Each dot represents the baseline microbiome composition of one IBS participant. Color denotes CBT responder status and dots are sized by the fraction of the microbiome comprised of the *Bacteroides* genus (**A**) or Clostridiales order (**B**). **C** Microbial genera with statistically significant association with CBT responder status (*q*<.05) are shown. The *y* axis shows the log2 of the fold change between responders vs. non-responders (NR). Dot size is proportional to mean relative abundance across all samples. **D** Baseline relative abundances (median scaled) in feces of the neurotransmitters serotonin, dopamine, and histamine. Lines indicate medians. * *p*<.05 by Mann–Whitney *U* test. e Non-metric multidimensional scaling analysis (NMDS) (stress=0.20) of global metabolomics profiles. Color denotes CBT responder status. *P* value calculated by Adonis, adjusting for sex and bowel habit subtype

A subgroup of 34 subjects underwent fecal sampling for microbiome analysis by 16S rRNA gene sequencing. This included 22 CBT responders (65%) and 12 non-responders (35%), who did not differ by clinical measures or baseline stool consistency as assessed by the Bristol Stool Scale (Table S2). Microbial alpha diversity measures of richness, evenness, and phylogenetic diversity did not significantly differ between responders and non-responders, while beta diversity analysis demonstrated a significant difference by responder status (*p*=.016) (Figs. 1, 5A). Responders had increased *Roseburia*, *Lachnobacterium*, and unclassified Lachnospiraceae (all members of the Clostridiales order) and decreased *Bacteroides*, *Parabacteroides*, and *Prevotella* (all members of the Bacteroidales order) compared to non-responders (Fig. 1C). The inverse relationship between the Clostridiales order and *Bacteroides* (the predominant genus enriched in non-responders) was apparent in the principal coordinates analysis plot, in which a gradient of Clostridiales (with a *Bacteroides* gradient in the reverse direction) separates baseline samples (Fig. 1A, B). Baseline severity as defined by IBS-SSS was not significantly associated with microbial diversity or composition (Figure S1).

#### Metabolomics

Fecal targeted and untargeted metabolomics analysis was performed on the 34 subjects to investigate the functional characteristics of the baseline microbiota associated with CBT response. Given the enrichment in responders of *Roseburia*, known to be a source of short-chain fatty acids such as butyrate, fecal short-chain fatty acid concentrations were measured. No significant differences were observed in 9 short-chain fatty acids between CBT responders and non-responders (Figure S2). The abundances of microbiome-derived neurotransmitters such as serotonin, norepinephrine, GABA, dopamine, and histamine were then assessed. Of these, fecal serotonin levels were increased in responders compared to non-responders (*p*=.03) (Fig. 1C). Norepinephrine and GABA were not detected, and there was no difference in dopamine or histamine between responders and non-responders. Expanding the analysis to the remaining fecal metabolites, there was no significant overall difference in global metabolomics profile between CBT responders and non-responders (Fig. 1D). Differential abundance testing did not reveal any metabolites with a statistically significant association with CBT response after correcting for multiple hypothesis testing, though 55 metabolites were nominally significant (Table S3).

#### Diet

We assessed baseline dietary intake for the 34 subjects who underwent fecal sampling using a food frequency questionnaire. CBT responders had lower carbohydrate intake, higher total fat and monounsaturated fat intake, and a trend towards increased fiber intake compared to non-responders (Table S4). However, there were no significant differences in intake of 32 food groups or 122 individual nutrients, including tryptophan.

#### Classifiers to predict CBT response

**Fig. 2:**
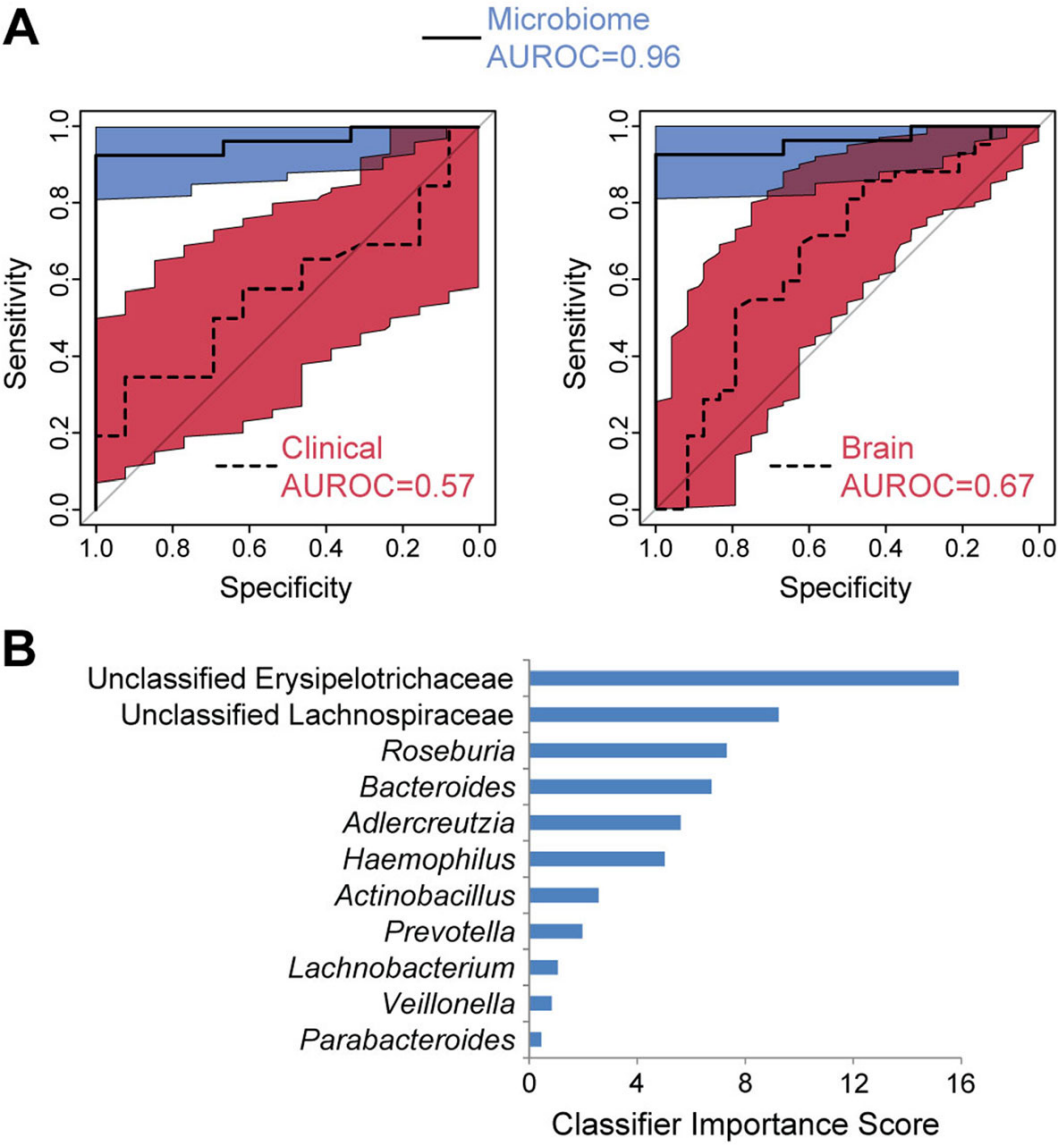
Classifiers derived from baseline fecal microbiota profiles outperformed those based on clinical/demographic and neuroimaging data to predict CBT response. **A** Receiver operating characteristic curves of random forest classifiers for CBT response constructed from differentially abundant microbial genera, baseline clinical/demographic data (left panel), or brain data (right panel). The 95% confidence intervals are represented as colored regions surrounding these curves (blue=microbiome, red=clinical/demographics or brain). **B** Importance scores for the 11 microbial genera in the random forests classifier

Random forest classifiers were created from baseline clinical data, brain features, or microbial taxa and assessed by 5-fold cross-validation. Of these, only the microbiome classifier had high accuracy to predict CBT response (AUROC 0.96). In contrast, a classifier constructed from demographic traits and baseline clinical measures had AUROC 0.57. Classifiers constructed using differentially abundant resting-state connections, FA, or ADC clusters had AUROC of 0.66, 0.51, and 0.49, respectively; when all brain datasets were combined, the resulting classifier had AUROC of 0.67. The increased accuracy of the microbiome classifier was highly significant by bootstrap analysis (*p*=6×10^−5^ and *p*=.0001 compared to the clinical/demographic and combined brain classifiers, respectively; there was no significant difference between the clinical/demographic and brain classifiers) (Fig. 2A). The taxa contributing most to microbiome classifier accuracy were unclassified Erysipelotrichaceae, unclassified Lachnospiraceae, *Roseburia*, and *Bacteroides* (Fig. 2B).

### Responders show Bacteroides expansion and distinct brain changes after CBT compared to non-responders

Post-treatment changes in clinical, neuroimaging, and microbiome parameters were compared between responders and non-responders to characterize the differential effects of CBT on the brain-gut-microbiome axis in responders.

#### Clinical changes post-CBT

Following the intervention, responders among the 84 subjects with neuroimaging data had a significant decrease in abdominal pain unpleasantness (Gracely, *p*=1×10^−6^), IBS symptom intensity (Gracely, *p*=4×10^−10^), negative mood ratings (POMS, *p*=.002), and perceived stress (PSS, *p*=1×10^−5^) compared to non-responders (Table S5). In addition, responders had a significant increase in IBS self-efficacy (*p*=2×10^−14^) and positive mood ratings (*p*=.0001). Although not as high and widespread as in the responders, the non-responders also had a significant increase in IBS self-efficacy (*p*=.001), and a decrease in IBS symptom intensity (*p*=.03).

#### Brain functional connectivity changes post-CBT

**Fig. 3:**
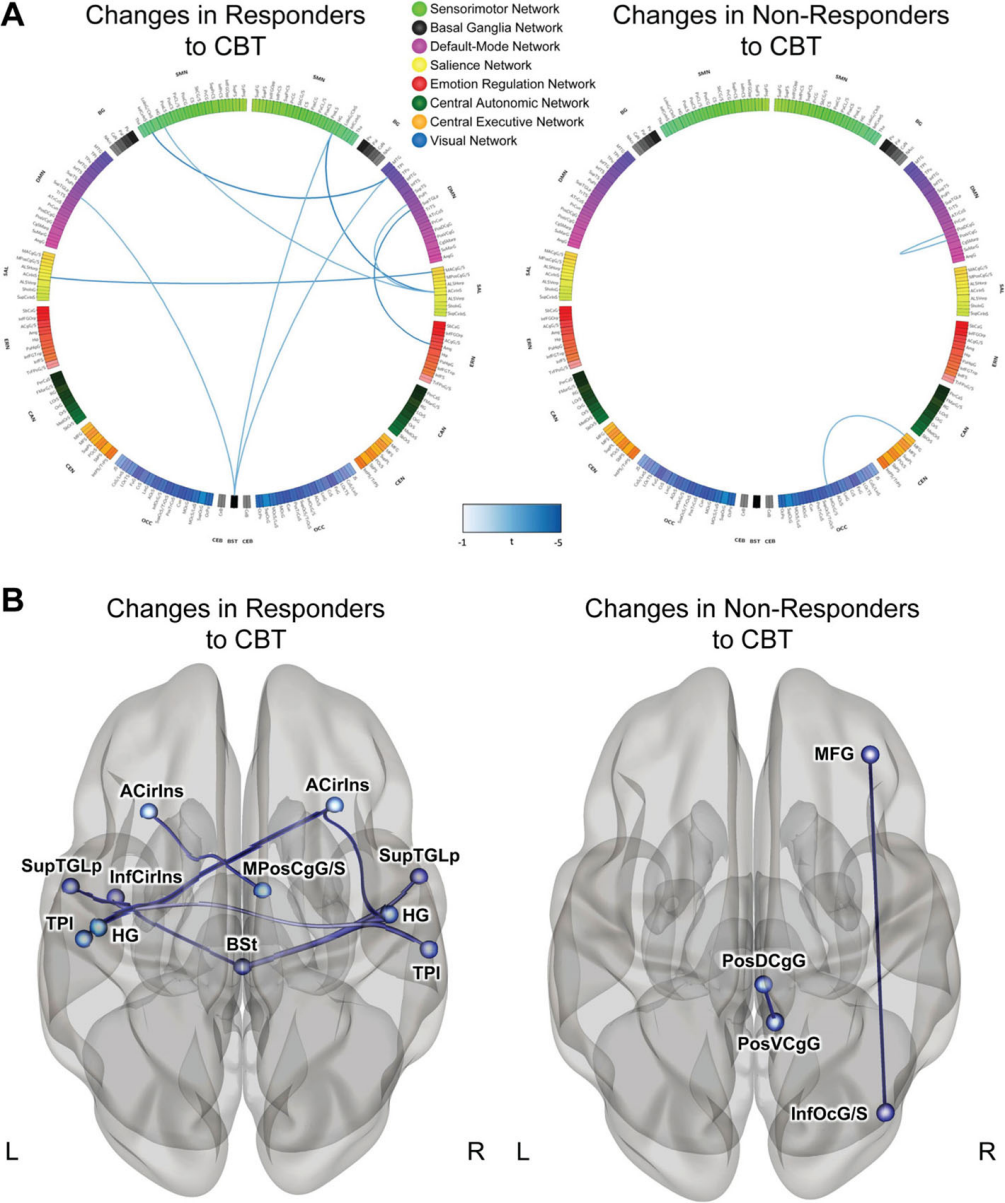
Changes in functional connectivity in responders and non-responders to CBT. **A** Connectograms demonstrating pair-wise connectivity differences in responders and non-responders to CBT. Significant decreases in connectivity between brain regions are denoted by blue lines connecting the regions (color intensity indicates magnitude of effect). There were no significant increases in connectivity. SMN: sensorimotor network, BG: basal ganglia, DMN: default mode network, SAL: salience network, ERN: emotion regulation network, CAN: central autonomic network, CEN: central executive Network, OCC: occipital/visual network. **B** Regions that significantly differed between responders and non-responders to CBT. Responders to CBT: ACirIns (anterior insula/anterior segment of the circular sulcus of the insula), MPosCgG/S (anterior mid-cingulate cortex), InfCirIns (anterior insula/inferior segment of the circular sulcus of the insula), SupTGLp (lateral aspect of the superior temporal gyrus), HG (Heschl’s gyrus), TPI (planum temporale), BSt (brainstem). Non-responders to CBT: MFG (middle frontal gyrus), PosDCgG (dorsal posterior cingulate cortex), PosVCgG (ventral posterior cingulate cortex), InfOcG/S (inferior occipital gyrus and sulcus)

Following CBT, responders showed a decrease in connectivity between multiple regions associated with specific brain networks, including the sensorimotor, default mode, salience, and emotion regulation networks (Table 1, Fig. 3). They also exhibited decreases in connectivity between regions associated with the brainstem and default mode and sensorimotor networks (all *q*<.05). Non-responders showed a decrease in connectivity between two links, one within the posterior cingulate (default mode network, *q*=.047) and one between the middle frontal gyrus (central executive network) and inferior occipital gyrus and sulcus (occipital lobe, *q*=.047). No increases in connectivity were observed for anyone undergoing CBT.

**Table 1:** CBT-related changes in resting-state functional connectivity in responders and non-responders

	Analysis Unit					
	*t* statistic	df	*p* value	*q* value	Cohen’s *d*	Interpretation
Responders to CBT						
Right anterior transverse temporal gyrus (Heschl’s Gyrus)—right anterior insula (anterior segment of the circular sulcus of the insula)	−4.07	117	.0001	.014	−0.75	Decrease after CBT
Left anterior insula (inferior segment of the circular sulcus of the insula)—right planum temporale	−4.00	117	.0001	.018	−0.74	Decrease after CBT
Left anterior insula (left anterior segment of the circular sulcus of the insula)—right aMCC	−3.80	117	.0002	.038	−0.70	Decrease after CBT
Right amygdala—right lateral aspect of the superior temporal gyrus	−3.79	117	.0002	.040	−0.70	Decrease after CBT
Right anterior insula (anterior segment of the circular sulcus of the insula)—right planum polare of the superior temporal gyrus	−3.59	117	.0005	.040	−0.66	Decrease after CBT
Brainstem—right planum temporale	−3.44	117	.0008	.044	−0.64	Decrease after CBT
Brainstem—right anterior transverse temporal gyrus (Heschl’s Gyrus)	−3.40	117	.0009	.044	−0.63	Decrease after CBT
Brainstem—left lateral aspect of the temporal gyrus	−3.36	117	.001	.044	−0.62	Decrease after CBT
Right anterior insula (anterior segment of the circular sulcus of the insula)—left anterior transverse temporal gyrus (Heschl’s gyrus)	−3.42	117	.0009	.048	−0.63	Decrease after CBT
Non-responders to CBT						
Right dPCC—right vPCC	−3.74	117	.0003	.047	−0.69	Decrease after CBT
Right inferior occipital gyrus and sulcus—right middle frontal gyrus	−3.74	117	.0003	.047	−0.69	Decrease after CBT

*Abbreviations*: *df* degrees of freedom, *CBT* cognitive behavioral therapy, *aMCC* anterior mid-cingulate cortex, *dPCC* dorsal posterior cingulate cortex, *vPCC* ventral posterior cingulate cortex

#### White matter integrity changes post-CBT

**Fig. 4:**
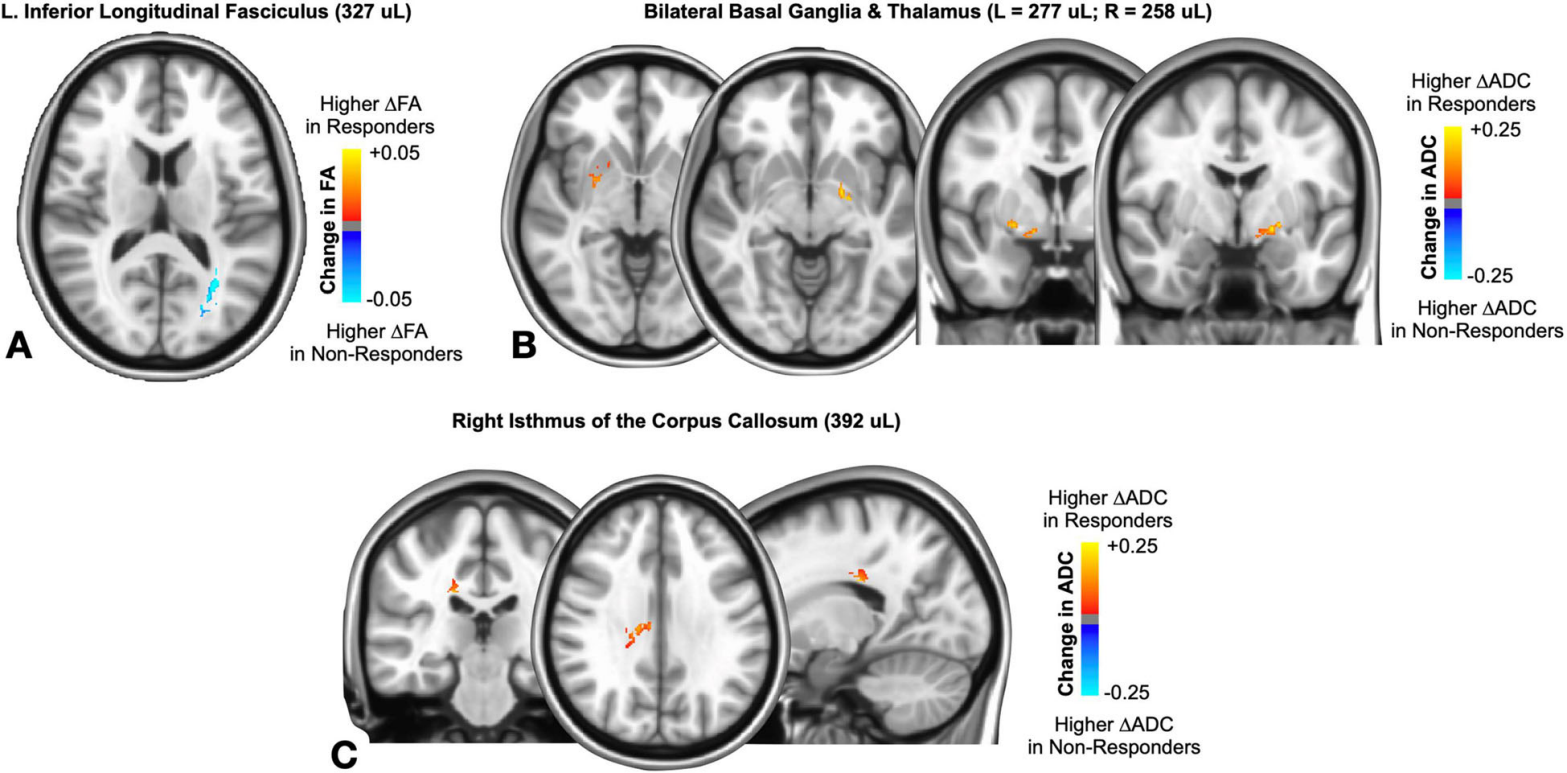
CBT responders had distinct changes in white matter integrity compared to non-responders. **A** Colored areas indicate regions within the left inferior longitudinal fasciculus that had a significant change in FA after CBT. Color corresponds to relative difference in FA change between CBT responders and non-responders. **B**, **C** Colored areas indicate regions of the bilateral basal ganglia and anterior thalamus (**B**) and isthmus of the corpus callosum (**C**) that had a significant change in ADC after CBT. Color corresponds to relative difference in ADC change between CBT responders and non-responders

A significantly higher change in FA was observed in CBT non-responders within the left inferior longitudinal fasciculus (Fig. 4A; cluster size = 327 μL). Additionally, we observed a significantly higher ADC in CBT responders bilaterally within areas encompassing the basal ganglia and anterior thalamus (Fig. 4B; left cluster size = 277 μL, right cluster size = 258 μL) as well as the isthmus of the corpus callosum (Fig. 4C; cluster size = 392 μL) in CBT responders compared with non-responders.

#### Microbiome and metabolomics changes post-CBT

**Fig. 5:**
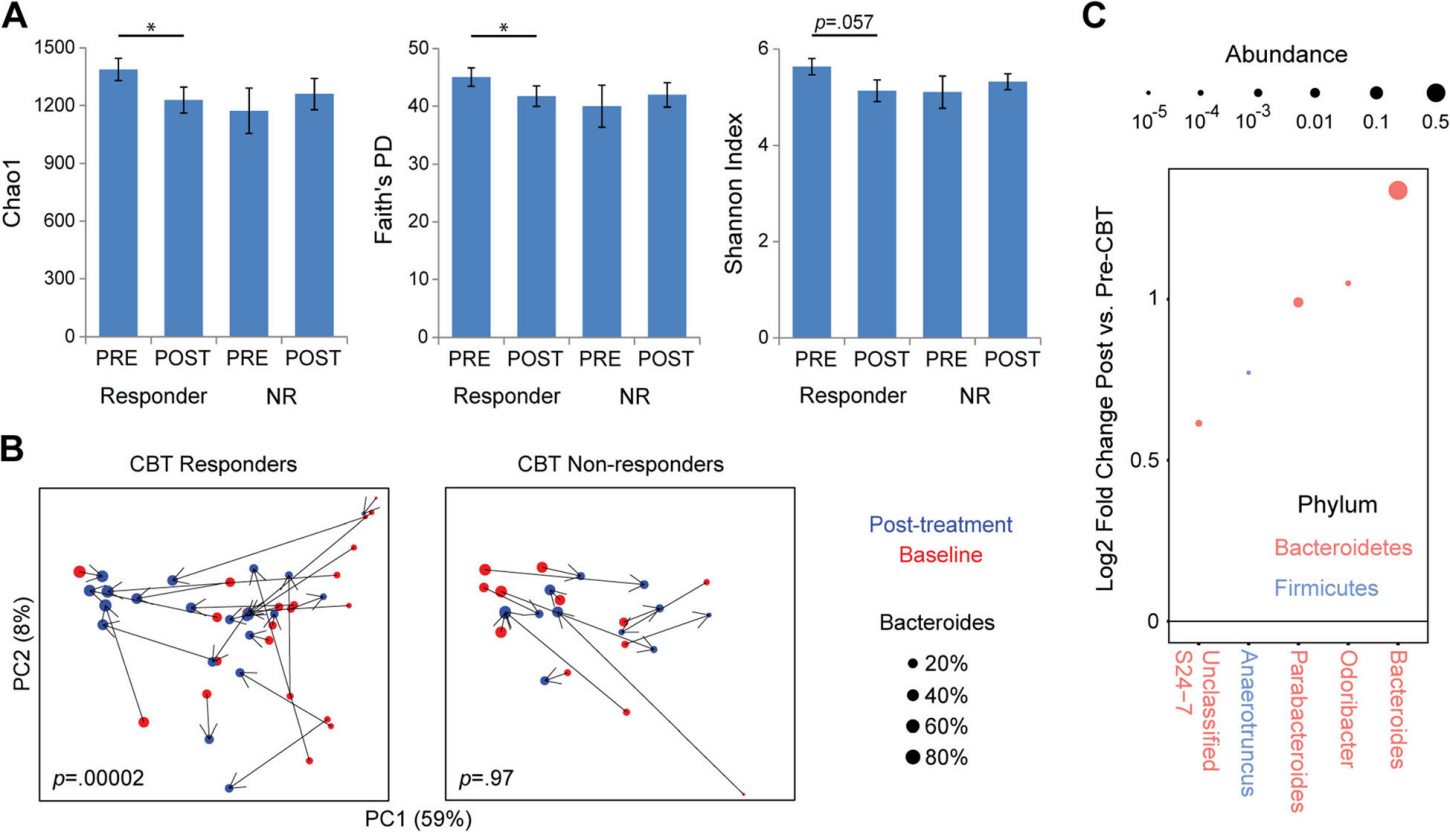
CBT responders have altered intestinal microbiome composition after CBT characterized by *Bacteroides* expansion. **A** Fecal microbial alpha diversity is shown for CBT responders and non-responders (NR) at baseline (PRE) and after CBT (POST). Three metrics are used: Chao1 index (richness), Faith’s phylogenetic diversity (PD), and Shannon index (richness and evenness). * *p*<.05. **B** Principal coordinates analysis of 16S rRNA sequence data before and after CBT, stratified by CBT response status. Each dot represents a sample, colored by time point (red=baseline, blue=post-CBT) and sized by *Bacteroides* abundance. Arrows connect samples from the same participants, with post-treatment indicated by the arrowhead. *P* values calculated by Adonis, adjusting for participant. **C** Microbial genera with statistically significant association with CBT responder status (*q*<.05) are shown. The *y* axis shows the log2 of the fold change between responders vs. non-responders. Dot size is proportional to mean relative abundance across all samples

Responders among the 34 subjects that underwent fecal sampling were found after CBT to have statistically significant post-treatment decreases in richness and phylogenetic diversity, whereas non-responders analyzed separately had no change in microbial diversity after treatment (Fig. 5A). Responders also demonstrated a highly significant shift in microbial composition (*p*=2×10^−5^) whereas non-responders showed no overall change in microbial composition (Fig. 5B). Differential abundance testing revealed five genera that were enriched in responders after CBT, including *Bacteroides*, *Odoribacter*, *Parabacteroides*, *Anaerotruncus*, and unclassified S24-7 (Fig. 5C). Of these, *Bacteroides* was the most abundant and had the greatest magnitude of change (2.5 fold increase, *q*=4×10^−5^), resulting in conversion of many patients to *Bacteroides*-predominant microbiota by the end of treatment (Fig. 5B). Metabolomics profiles did not differ between baseline and end of treatment for both groups, and no differentially abundant metabolites or short-chain fatty acids were identified in CBT responders after treatment when adjusting for multiple hypothesis testing (Table S7, Figure S2).

#### Diet and stool consistency post-CBT

CBT responders among the 34 subjects that underwent microbiome analysis did not have any significant changes in dietary macronutrient content or intake of specific food groups and nutrients as assessed by a food frequency questionnaire administered 2 weeks after completion of CBT (Table S8). There were no significant changes in stool consistency as assessed by the Bristol Stool Scale in either responders or non-responders (Figure S3).

### Changes in brain parameters in CBT responders correlate with clinical outcomes

#### Brain functional connectivity (Table 2)

In responders, decreased connectivity between key nodes of the salience network (right anterior mid-cingulate cortex and left anterior insula) was associated with higher positive mood ratings (POMS; *r*_*(49)*_=.29, *q*=.036). Reduced connectivity between the brainstem and left *superior temporal gyrus* was associated with higher positive mood (POMS; *r*_*(49)*_=.36, *q*=.036) and lower negative mood (POMS; *r*_*(49)*_=−.32, *q*=.042). Decreased connectivity between the right anterior INS and right *superior temporal gyrus* was associated with lower unpleasantness rating of abdominal pain (Gracely; *r*_*(49)*_=−.33, *q*=.036).

**Table 2:** Associations between changes in resting-state functional connectivity and DTI after CBT with clinical measures in responders

Changes in functional connectivity after CBT are associated with clinical measures in responders					
Dyad with reduced connectivity	Clinical measure	*r*	*df*	*p* value	*q* value
Right aINS—right PoPl	Unpleasantness rating of IBS symptoms	−.33	49	.02	.036
Left aINS—right aMCC	POMS positive score	.29	49	.039	.036
Brainstem—left SupTGLp	POMS positive score	.36	49	.009	.036
Brainstem—left SupTGLp	POMS negative score	−.32	49	.022	.042
Changes in DTI after CBT are associated with clinical measures in responders					
DTI measure—cluster	Clinical measure	*r*	*df*	*p* value	*q* value
FA—inferior longitudinal fasciculus	PANAS positive affect	.38	41	.012	.04
ADC**—**brainstem	POMS—positive score	.41	42	.006	.03

*Abbreviations*: *r* partial Pearson’s correlation (controlling for age and sex), *df* degrees of freedom, *p p* value, *CBT* cognitive behavioral therapy, *IBS* irritable bowel syndrome, *aMCC* anterior mid-cingulate cortex, *aINS* anterior insula, *SupTGLp* lateral aspect of the superior temporal gyrus, *PoPl* planum polare of the superior temporal gyrus, *FA* fractional anisotropy, *ADC* apparent diffusion coefficient, *POMS* Profile of Mood States, *PANAS* Positive and Negative Affect Schedule

#### White matter integrity (Table 2)

In responders, CBT-associated decreases in FA values in the inferior longitudinal fasciculus were significantly associated with increases in positive affect (PANAS, positive, *q*=.040), and higher ADC in the brainstem was associated with higher positive mood scores (POMS positive, *q*=.040).

### CBT-induced brain changes correlate with the microbiome in responders

#### Brain functional connectivity

Alterations in neuroimaging parameters following CBT were correlated with microbiome shifts in subjects who underwent fecal sampling. In responders, the increases in the abundance of two taxa (*Bacteroides*, *r*_*(27)*_=−.63, *q* =.022; unclassified S24-7, *r*_*(27)*_=−.55, *q*=.041) were negatively associated with reduced resting-state connectivity between the brainstem and a region in the temporal network (Table 3).

**Table 3:** Associations between resting-state functional connectivity after CBT with abundance of gut microbes in responders

Changes in functional connectivity after CBT are associated with abundance of gut microbes in responders					
Microbial genera	Connectivity—dyad	*r*	*df*	*p* value	*q* value
*Bacteroides*	Brainstem—left lateral aspect of the temporal gyrus	−.63	27	.011	.022
Unclassified S24-7	Brainstem—right planum temporale	−.55	27	.041	.041

*Abbreviations*: *r* partial correlation (controlling for age and sex), *df* degrees of freedom, *p p* value, *CBT* cognitive behavioral therapy

#### White matter integrity

No statistically significant correlations were observed between the microbiome and white matter integrity in responders.

## Discussion

We demonstrate that a positive clinical response in CBT-treated IBS patients is associated with changes in functional and structural connectivity of brain networks, as well as changes in gut microbiota, compared to CBT-treated patients who did not achieve clinical response. Additionally, we found that a classifier including 11 microbial genera was able to predict treatment outcome with high accuracy. To our knowledge, this is the first demonstration of an association between treatment outcomes and changes in brain and gut microbiota for any IBS therapy (e.g., pharmacological, dietary). These findings support the concept that even though CBT is considered a psychological treatment that teaches cognitive skills for remediating perceptual biases, the symptom changes it induces may occur via modulation of brain-gut-microbiome interactions which influence IBS pathophysiology and visceral symptom generation.

Although responders and non-responders to CBT did not differ on any clinical or behavioral parameters pre-treatment, minor baseline differences were observed in functional connectivity and significant baseline differences were observed in the relative abundances of gut microbes. This suggests that CBT responders have pre-existing differences in central and peripheral components of the brain-gut-microbiome axis relative to non-responders that confer increased sensitivity to the effects of CBT on GI symptomology. Compared to CBT non-responders, responders at baseline showed greater functional connectivity between regions of the central autonomic network and the emotional regulation network. Based on previously reported functional and anatomical connectivity brain differences between IBS and healthy controls, these findings suggest that IBS-characteristic brain changes were not more pronounced in CBT responders versus CBT non-responders at baseline and that the observed brain differences between the two groups were only more pronounced after the CBT intervention [21, 32, 33].

While there is currently no agreement on the existence of IBS characteristic alterations in the gut microbiota, evidence for possible microbial-based subgroups has been reported [9, 10]. The current findings add to this concept, demonstrating that microbiota could identify subsets of IBS-patients with differential responsiveness to CBT. Responders had increased levels of several members of the Clostridiales order (*Roseburia*, *Lachnobacterium*, unclassified Lachnospiraceae) and decreased levels of members of the Bacteriodales order (*Bacteroides*, *Parabacteroides*, *Prevotella*) compared to non-responders, resulting in gradients of Clostridiales and *Bacteroides* separating the baseline samples. Strikingly, a classifier using 11 differentially abundant genera showed high accuracy to predict CBT response from baseline microbiota. This contrasted with the weak predictions that could be made from clinical or neuroimaging data and suggests that microbial composition could serve as an accurate biomarker for biological pathways in IBS symptom generation that are modulated by CBT if these findings are validated in an independent cohort. Diet is one potential factor contributing to baseline microbial differences between CBT responders and non-responders. In our study, we found that responders reported decreased fraction of energy intake from carbohydrates and increased fraction from unsaturated fats. Dietary pattern, however, was not affected by treatment and is therefore unlikely to explain changes in clinical and biological parameters.

The strong association of the baseline microbiome with CBT response suggests that signals from the gut microbiome could potentiate or suppress the effects of CBT on target brain regions. Clostridiales encompasses many spore-forming microbes which have previously been shown to induce intestinal serotonin synthesis and release, representing a potential link between the gut microbiome and CBT responsiveness [34]. Fecal metabolomics analysis showed increased baseline serotonin levels in responders, consistent with a greater tonic luminal release of serotonin into the gut lumen or potentially serotonin production by luminal bacteria [35]. Acute tryptophan depletion has previously been shown to induce IBS-like symptoms and brain changes in healthy controls, supporting a role of serotonin as a modulator of the BGM axis [36]. Differential abundance testing did not reveal any other metabolites with a statistically significant association with CBT response after correcting for multiple hypothesis testing. However, 55 metabolites were nominally significant, including four—delta-tocopherol, gamma-tocopherol/beta-tocopherol, chenodeoxycholate, and m-tyramine—related to metabolites previously shown to induce serotonin release by cultured Enterochromaffin cells in vitro [34]. A recent study by E. Hsiao’s group in mouse models demonstrated that different interventions aimed at increasing intestinal serotonin result in an increase of spore-forming Clostridia, including Clostridiales and Lachnospiraceae [37]. These findings, together with the earlier study from their lab, support a circular interaction between Clostridiales in the gut, host epithelial serotonin production and release, and serotonin-induced increase in Clostridia abundance, in which Clostridiales promote their own community membership in the gut microbiota.

Following treatment, and similar to the results obtained in the parent study, responders showed highly significant improvements in several clinical parameters, including IBS symptom severity and its constituent processes (abdominal pain, life satisfaction, bowel dissatisfaction). These clinical improvements were accompanied by functional and structural brain changes as responders showed a reduction in the connectivity between multiple cortical networks, including the sensorimotor, default mode, salience, and emotion regulation networks. A previous report on the effectiveness of CBT in patients with multiple chronic pain conditions had shown a similar reduction in the functional connectivity between regions of the default mode and emotion regulation networks which was associated with an increase in functional connectivity between the basal ganglia network and the right somatosensory cortex [38]. Similar to our study, they showed that these brain changes were associated with improvements in clinical and behavioral measures. In the current study, a reduction in the connectivity within the salience network and a reduction in brainstem connectivity with the left superior temporal gyrus were both associated with more positive mood ratings and reduced negative affective ratings of IBS symptoms. CBT responders also showed significant changes in white matter integrity, with greater change in ADC within the basal ganglia and anterior thalamic regions, and increased ADC values in white matter tissue near the right somatosensory regions, compared to non-responders. This CBT-associated normalization of microstructural brain changes in the basal ganglia and thalamic areas were associated with increase in positive mood and positive affect. The IBS patients with improved symptoms following CBT had decreased connectivity between the bilateral Heschl’s gyrus and the anterior insula, a key hub of the salience network. The Heschl’s gyrus is known as the primary auditory cortex which would be highly active during a resting-state fMRI scan due to the noise present in the scanner. Lower connectivity with the anterior insula would suggest that the improvers are perceiving the stimulus as less salient. This could be attributed to a number of reasons such as better mood, better coping skills learned from the CBT intervention, and better health outcomes overall. Future research would need to investigate the role of the interaction between the salience and sensorimotor network to get a better understanding of these results.

Treatment-induced changes were also seen in the diversity and relative abundances of the gut microbiota. While there was a decrease in richness and phylogenetic diversity, five genera were enriched in responders, with *Bacteroides* being the most abundant. Thus, while low levels of *Bacteroides* abundance prior to treatment were predictive of a response, CBT increased the abundance of this genus, resulting in a conversion of many patients to *Bacteroides*-predominant microbiota. The reduction in microbial diversity in CBT responders may reflect displacement of enteric microbes by the expanded *Bacteroides* population. As CBT only targets brain and behavioral mechanisms and there was no evidence of CBT-associated alterations in dietary intake in responders, the change in microbiome is likely secondary to effects of CBT on the brain. Further supporting the role of the brain in these microbial abundance changes, the observed functional and structural brain changes were associated with an increase in *Bacteroides* and unclassified S24-7, a family of microbes belonging to the order of Bacteroidales. These findings suggest that a gut microbial community state (Clostridiales high, *Bacteroides* low) is responsive to top-down signals from the brain affected by the cognitive skills learned through CBT, resulting in conversion to an alternate state enriched in *Bacteroides*. While the association of this microbial change with clinical improvement suggests its importance in symptom generation, baseline microbial composition was not significantly associated with severity by IBS-SSS. This argues that microbial change alone is insufficient for protection against symptom generation in IBS and emphasizes the importance of brain alterations for CBT response.

The strengths of this study are its prospective, longitudinal design and incorporation of both multimodal neuroimaging and multi-omics microbiome assessment to evaluate the BGM axis. Although the sample size of this study was sufficient to identify robust changes in symptoms, brain parameters, and gut microbial abundances, it limited our ability to identify statistically significant differences between responders and non-responders in microbial metabolites. Other limitations include the lack of a reference healthy control population, validation cohort, or long-term follow-up to evaluate the durability of the microbiome and neuroanatomical changes in CBT responders.

## Conclusions

This study demonstrates for the first time that a brief non-drug, non-dietary intervention that teaches information processing skills can modulate key components of the brain-gut-microbiome axis in IBS patients. Moreover, their likelihood of treatment response could be predicted from baseline microbiota composition, raising the possibility that CBT-responsive IBS patients can be identified in clinical practice using microbial biomarkers. The observed changes in brain, gut microbes and symptoms in patients who responded to this brain directed therapy supports the role of alterations in the brain-gut-microbiome axis in IBS, and is most consistent with an important influence of the brain on the gut microbiome. Larger studies are needed to characterize the functional correlates of gut microbial changes and to identify distinct subtypes of IBS patients for whom brain- and gut-directed therapies are most effective.

## Supplementary Information

**Additional file 2.** Short-chain fatty acids.

**Additional file 3.**

## Data Availability

The sequencing data supporting the conclusions of this article are available in the NCBI Bioproject repository, PRJNA736955 (https://www.ncbi.nlm.nih.gov/bioproject/736955). The untargeted metabolomics data have been deposited in the Metabolomics Workbench repository under ID 2820 (https://www.metabolomicsworkbench.org). Short-chain fatty acid data are included with this article as a supplementary data file. The deidentified and raw neuroimaging data are available through the NIH funded PAIN (pain and interoception imaging network) data repository (https://www.painrepository.org/repositories), which includes multimodal MRI scans in healthy controls and various chronic pain conditions. Access to the data is based on membership which is based on a collaborative principle.
