# Supplementary material for: Cognitive behavioral therapy for irritable bowel syndrome induces bidirectional alterations in the brain-gut-microbiome axis associated with gastrointestinal symptom improvement
Source: Microbiome. 2021 Nov 30;9:236. doi: 10.1186/s40168-021-01188-6 (PMC8630837; doi:10.1186/s40168-021-01188-6)
Supplement: Supplementary file 2 — Additional file 2. Short-chain fatty acids. [file 40168_2021_1188_MOESM2_ESM.docx]

**SUPPLEMENTARY Methods**

**Clinical/Behavioral Measures**

The *Gracely Box scale* [1] is a validated visual analog scale (of length 20) for pain or discomfort in the areas of intensity (sensory) and unpleasantness (affect). It was used to evaluate IBS symptoms in the last 24 hours and the same scale was used post resting state fMRI scan to any assess any bodily pain or discomfort experienced during the scanning. Greater scores were representative of greater IBS-related discomfort.

The *IBS Self-Efficacy scale* (IBS-SE) was adopted from the Headache Self-Efficacy Scale [2] and assesses the individual’s belief that they are able to prevent a moderately painful abdominal pain episode when confronted with precipitants of abdominal pain. The sum of the 25 items was used as a total self-efficacy score.

The *Pennebaker Inventory of Limbic Languidness (*PILL) [3] was used to measure general sensory perception, including visceral and somatic sensations. The PILL assesses the frequency of common physical sensational phenomena and symptoms, for instance skin sensations or symptoms (e.g. itchiness, sensitivity, rashes, acne), vision or hearing and eye or nose issues, pain, digestive, respiratory or cardiac events. The score is composed of the sum of categorical scores assigned to a five-point Likert scale from “never experienced the symptom” to experiencing the symptom “more than once a week”. For each of the 54 items, 1 point is given if the participant marks the item as occurring about at least once a month, yielding a maximum possible score of 54.

The *Profile of Mood States* (POMS) questionnaire [4] consists of 65 words/statements which describe feelings people have. Each word/statement is scored on a 5-point Likert scale (0-4) with 0 = Not at All, 1 = A Little, 2 = Moderately, 3 = Quite a Lot, 4 = Extremely. A Total Mood Disturbance (TMD) score is then calculated by summing the totals from the negative subscales (e.g. anger, depression) and subtracting the totals of the positive subscales (e.g. vigor, esteem-related affect).

The *Perceived Stress Scale (*PSS) [5] measures the extent to which ongoing life events over the last month are described as unpredictable, overwhelming, and uncontrollable using a 5-point Likert scale (0-4) with 0 = Never, 1 = Almost Never, 2 = Sometimes, 3 = Fairly Often, 4 = Very Often.

**Statistical Testing of Behavioral/Clinical Variables**

To determine differences in behavioral/clinical variables before and after CBT, a paired t-test was used with significance being set at α =.05. Cohen’s d effect size values were also computed to determine the magnitude of the difference between standardized means before and after CBT. Cohen’s *d* values of 0.2 indicate a small effect, 0.5 a medium effect, 0.8 a large effect, and 1.20 a very large effect.

**MRI Acquisition**

Whole-brain structural, functional (resting-state) and diffusion tensor imaging data was acquired using the following parameters: Acquisition parameters for high resolution T1-weighted images were as follows: echo time/repetition time (TE/TR) = 3.01/2300ms, field of view (FOV) = 256mm, slice thickness = 1mm, 176 slices, 256 x 240 acquisition matrix, voxel size = 1mm^3^. Functional resting-state scans were acquired with eyes closed and an echo-planar sequence with the following parameters: TE/TR: 28/2000ms, flip angle = 77 degrees, scan duration = 10 min, FOV = 220mm, slices = 300, slice thickness = 4.0mm, voxel-size = 3.4 x 3.4 x 4.5 mm, and slices were obtained with whole brain coverage. Diffusion tensor scans were acquired with the following parameters: 64 directions with b = 1000s/mm^2^, along with 1 b = 0 s/mm^2^ image, TE/TR = 88/13300ms, FOV = 230mm, slice thickness = 2mm (2.2mm interslice gap), voxel size = 1.98 x 1.98 x 2.2 mm, and a matrix of 116 x 116. All scans were acquired twice, once before and after the CBT or education intervention.

**MRI Processing**

*Structural Processing.* Preprocessing and quality control of structural images were done using Statistical Parametric Mapping 12 (SPM12). All structural images were skull stripped, segmented, then normalized to the MNI T1 template. This created normalized T1 images for every participant along with segmented images (gray matter, while matter and cerebral spinal fluid [CSF]) in normalized space.

*Structural Image Parcellation.* T1-image segmentation and cortical and subcortical regional parcellation were conducted using FreeSurfer v.6.0 [6] following the nomenclature described in the Destrieux and Harvard-Oxford subcortical atlas [7, 8]. The parcellation results in the labeling of 165 cortical regions, 74 bilateral cortical structures, 7 subcortical structures, the midbrain, and the cerebellum.

*Resting-State fMRI Processing.* Preprocessing and quality control of functional images was done using SPM-12 software and involved slice-time correction and motion correction for the six realignment parameters. If any motion was detected above 2 mm translation or 2˚ rotation, the scan, along with the paired structural scan was discarded. In order to robustly take account of the effects of motion, root mean squared (RMS) realignment estimates were calculated as robust measures of motion using publicly available MATLAB code from GitHub [9]. The resting state images were then co-registered to their respective anatomical T1 images. Each T1 image was then segmented and normalized to a smoothed template brain in Montreal Neurological Institute (MNI) template space. Each participant’s T1 normalization parameters were then applied to that participant's resting state image, resulting in an MNI space normalized resting state image. The resulting images were smoothed with 5mm3 Gaussian kernel. For each participant, a sample of the volumes was inspected for any artifacts and anomalies. Levels of signal dropout were also visually inspected for excessive dropout in a priori regions of interest.

*Functional Network Construction.* Functional brain networks were constructed using the CONN 17 toolbox [10] in MATLAB. Regions from the Destrieux [7] and Harvard-Oxford [8] Subcortical Atlases were entered as regions of interest (ROIs). To summarize, all pre-processed, normalized images taken before and after CBT were first corrected for noise using the automatic component-based noise correction (CompCor) method to remove physiological noise without regressing out the global signal [11]. Confounds for the six motion parameters along with their first-order temporal derivatives, along with confounds emerging from white matter and CSF, and first-order temporal derivatives of motion, and RMS values of the detrended realignment estimates were removed using regression. Although the influence of head motion cannot be completely removed, CompCor has been shown to be particularly effective for dealing with residual motion relative to other methods. The images were then put through a temporal band-pass filter between 0.008 – 0.09 Hz after regression in order to minimize the effects of low frequency drift and high frequency noise after CompCor regression. Connectivity matrices for each participant at both time points, consisting of all the parcellated regions in the Destreuix [7] and Harvard-Oxford [8] Subcortical Atlas (165 x 165), were then computed. This represents the association between two average temporal BOLD time series across all the voxels in each region. The final outputs for each participant consisted of a connectivity matrix between the 165 parcellated regions and was indexed by Fisher transformed Z correlation coefficients.

*DTI Processing.* DTI data were processed using methodology similar to previous studies [12-14]. Briefly, diffusion MRI scans were corrected for eddy currents and motion using the eddy correct functionality of the FSL Diffusion Toolbox (FDT) as part of FSL (FMRIB; Oxford, UK) [15]. Fractional Anisotropy (FA) and Apparent Diffusion Coefficient (ADC) images, both of which reflect the integrity of white matter, were calculated using the MRtrix package (Brain Research Institute, Melbourne, Australia, http://www.brain.org.au/software), and registered to the ICBM-DTI 1mm FA atlas using linear (12 direction via FSL FLIRT) and then nonlinear (via FSL FNIRT) registration on the FA images, the transforms of which were then applied to the ADC images. After registration to the atlas, white matter regions were segmented using an FA threshold of 0.3. Deep gray matter and subcortical regions including the thalamus, basal ganglia, and brainstem were also retained for subsequent analyses.

**Dietary Assessment**

The Diet History Questionnaire (DHQ) II was administered to subjects during the 4 week baseline period before initiation of CBT and at the post-treatment assessment performed 2 weeks after the end of CBT treatment. The one-month version of DHQ II with serving sizes was used. Subject responses were inputted into the Diet*Calc software for prediction of nutrient intake using the DHQII nutrient and food group database [16]. Significance of differences in macronutrient intake (estimated using USDA values) was assessed by the Mann-Whitney U test or Wilcoxon signed-rank test (when comparing baseline to post-CBT time points). Intake of 32 food groups was estimated using the MyPyramid Equivalents Database (MPED). Intake of 122 individual nutrients including individual amino acids, sugars, fatty acids, and vitamins was estimated using values from USDA and Nutrition Data System for Research (NDSR). General linear models implemented in limma were employed to assess for differences (controlling for sex and bowel habit subtype) in individual food groups and nutrients between responders and non-responders at baseline, and between baseline and post-CBT in responders and non-responders analyzed separately [17]. *P*-values were adjusted for multiple hypothesis testing using the Benjamini-Hochberg procedure.

**References**

1. Gracely RH, McGrath P, Dubner R: **Ratio scales of sensory and affective verbal pain descriptors**. *Pain* 1978, **5**(1):5-18.

2. American College of Gastroenterology Task Force on Irritable Bowel S, Brandt LJ, Chey WD, Foxx-Orenstein AE, Schiller LR, Schoenfeld PS, Spiegel BM, Talley NJ, Quigley EMM: **An evidence-based position statement on the management of irritable bowel syndrome**. *The American journal of gastroenterology* 2009, **104 Suppl 1**:S1-35.

3. Pennebaker JW: **Putting stress into words: Health, linguistic, and therapeutic implications**. *Behaviour Research and Therapy* 1993, **31**(6):539-548.

4. Mcnair DM, Lorr M, Droppleman LF: **Manual for the Profile of Mood States**. In*.* Toronto, ON: Multi-Health Systems Inc.; 1971.

5. Cohen S, Kamarck T, Mermelstein R: **A global measure of perceived stress**. *Journal of Health and Social Behavior* 1983, **24**(4):385-396.

6. Reuter M, Schmansky NJ, Rosas HD, Fischl B: **Within-subject template estimation for unbiased longitudinal image analysis**. *Neuroimage* 2012, **61**(4):1402-1418.

7. Destrieux C, Fischl B, Dale A, Halgren E: **Automatic parcellation of human cortical gyri and sulci using standard anatomical nomenclature**. *Neuroimage* 2010, **53**(1):1-15.

8. Lowe MJ, Dzemidzic M, Lurito JT, Mathews VP, Phillips MD: **Correlations in low-frequency BOLD fluctuations reflect cortico-cortical connections**. *Neuroimage* 2000, **12**(5):582-587.

9. Power JD, Mitra A, Laumann TO, Snyder AZ, Schlaggar BL, Petersen SE: **Methods to detect, characterize, and remove motion artifact in resting state fMRI**. *Neuroimage* 2014, **84**:320-341.

10. Whitfield-Gabrieli S, Nieto-Castanon A: **Conn: a functional connectivity toolbox for correlated and anticorrelated brain networks**. *Brain Connect* 2012, **2**(3):125-141.

11. Behzadi Y, Restom K, Liau J, Liu TT: **A component based noise correction method (CompCor) for BOLD and perfusion based fMRI**. *Neuroimage* 2007, **37**(1):90-101.

12. Ellingson BM, Mayer E, Harris RJ, Ashe-McNally C, Naliboff BD, Labus JS, Tillisch K: **Diffusion tensor imaging detects microstructural reorganization in the brain associated with chronic irritable bowel syndrome**. *Pain* 2013, **154**(9):1528-1541.

13. Woodworth D, Mayer E, Leu K, Ashe-McNalley C, Naliboff BD, Labus JS, Tillisch K, Kutch JJ, Farmer MA, Apkarian AV *et al*: **Unique Microstructural Changes in the Brain Associated with Urological Chronic Pelvic Pain Syndrome (UCPPS) Revealed by Diffusion Tensor MRI, Super-Resolution Track Density Imaging, and Statistical Parameter Mapping: A MAPP Network Neuroimaging Study**. *PLoS One* 2015, **10**(10):e0140250.

14. Woodworth DC, Dagher A, Curatolo A, Sachdev M, Ashe-McNalley C, Naliboff BD, Labus JS, Landis JR, Kutch JJ, Mayer EA *et al*: **Changes in brain white matter structure are associated with urine proteins in urologic chronic pelvic pain syndrome (UCPPS): A MAPP Network study**. *PLoS One* 2018, **13**(12):e0206807.

15. Jenkinson M, Beckmann CF, Behrens TE, Woolrich MW, Smith SM: **Fsl**. *Neuroimage* 2012, **62**(2):782-790.

16. Subar AF, Midthune D, Kulldorff M, Brown CC, Thompson FE, Kipnis V, Schatzkin A: **Evaluation of alternative approaches to assign nutrient values to food groups in food frequency questionnaires**. *Am J Epidemiol* 2000, **152**(3):279-286.

17. Ritchie ME, Phipson B, Wu D, Hu Y, Law CW, Shi W, Smyth GK: **limma powers differential expression analyses for RNA-sequencing and microarray studies**. *Nucleic Acids Res* 2015, **43**(7):e47.

**Supplementary Figures and Tables**


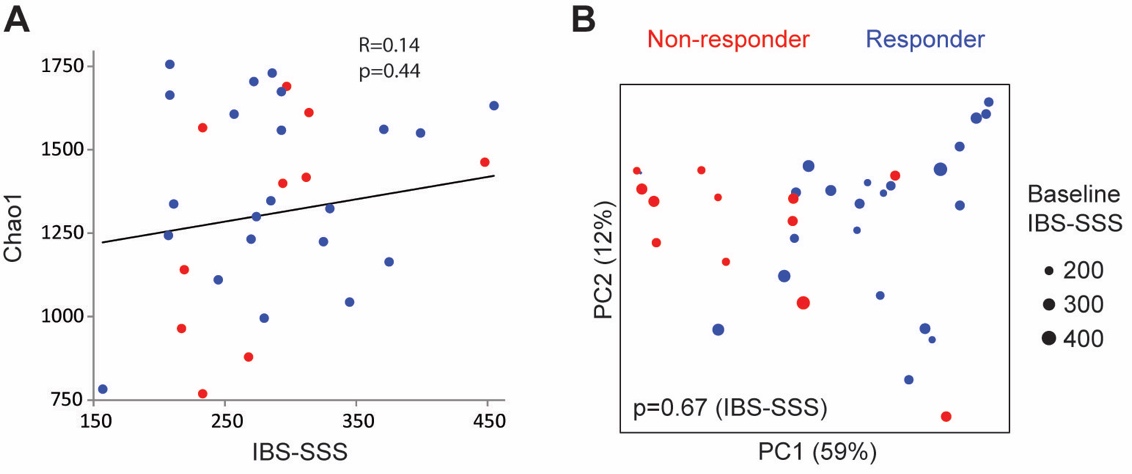


**Supplementary Figure 1. Baseline disease severity is not associated with fecal alpha or beta diversity.** (**A**) Scatter plot depicting baseline severity (measured by IBS-SSS) in relation to microbial richness (as assessed by the Chao1 index). Linear regression line is shown. (**B**) Principal coordinates analysis of 16S rRNA sequence data at baseline. Color denotes CBT responder status and dots are sized by severity (IBS-SSS). *P*-value calculated by Adonis, adjusting for sex and bowel habit subtype.**
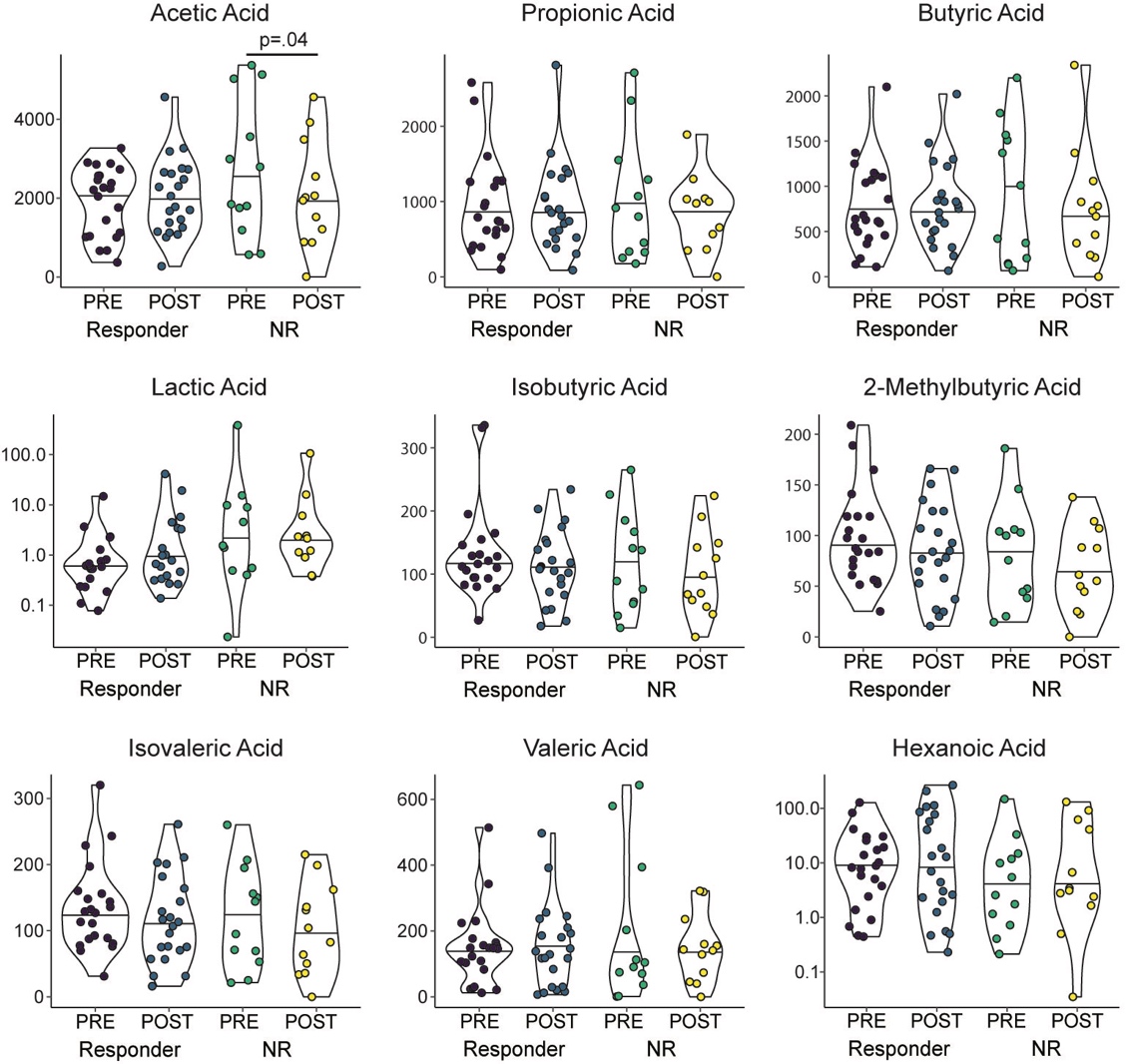
**

**Supplementary Figure 2. Fecal short chain fatty acids did not differ between responders and non-responders at baseline and did not change after CBT.** Fecal concentrations (µg/g feces) of nine short chain fatty acids are shown for CBT responders and non-responders (NR) at baseline (PRE) and post-CBT (POST). Significance of differences between responders and NR at baseline was assessed by the Mann-Whitney U test. Significance of differences between baseline and post-CBT was assessed by the Wilcoxon signed-rank test. The only nominally significant difference is shown in the figure.

**
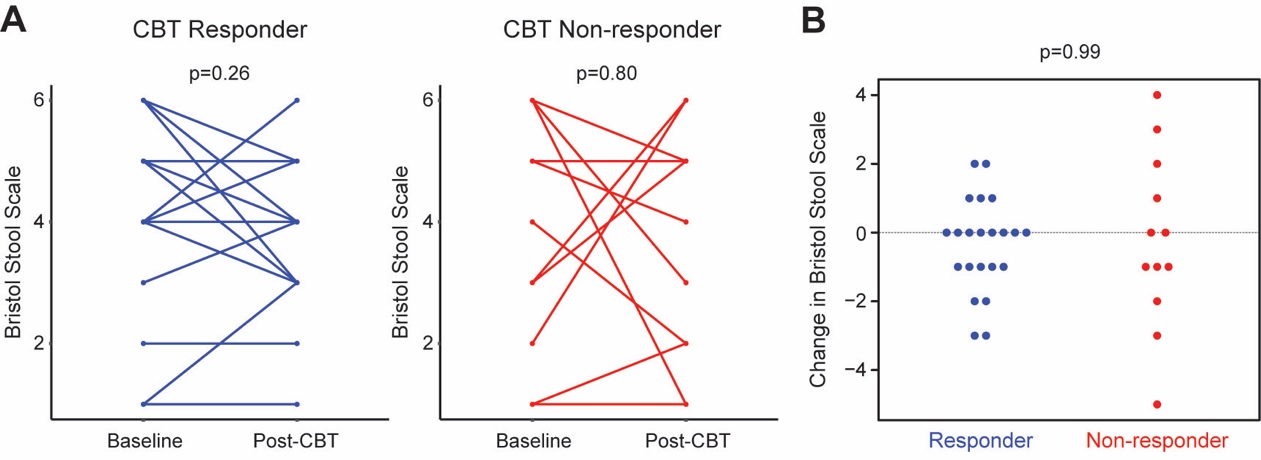
**

**Supplementary Figure 3. CBT was not associated with a significant change in stool consistency in either responders or non-responders.** (**A**) Line graphs showing baseline and post-CBT stool consistency by the Bristol Stool Scale for CBT responders and non-responders. *P*-values calculated by the Wilcoxon signed-rank test. (**B**) Dot graph showing the change in Bristol Stool Scale following CBT for CBT responders and non-responders. *P*-value calculated by the Mann-Whitney U test.

**Supplementary Table 1: Baseline differences in clinical measures between CBT responders and non-responders in neuroimaging and clinical outcomes analyses**

| Variable | Responder  (n = 58) | Non-Responder  (n = 26) | *P*-value | Cohen’s *d* |
| --- | --- | --- | --- | --- |
| Female sex, n (%) | 47 (81.0) | 23 (88.5) | .53 |  |
| Age, mean (SD) | 40.5 (14.5) | 38.2 (14.1) | .48 | 0.16 |
| BMI, mean (SD) | 25.6 (6.3) | 26.0 (7.7) | .80 | 0.06 |
| Gracely Scale, mean (SD) |  |  |  |  |
| Unpleasantness of IBS sxs | 10.7 (4.1) | 10.3 (3.2) | .58 | 0.12 |
| Intensity of IBS sxs | 12.2 (3.9) | 12.0 (4.5) | .86 | 0.05 |
| fMRI pain/discomfort ^a^ | 0.32 (0.47) | 0.38 (0.50) | .59 | -0.13 |
| IBS-SE, mean (SD) | 97.6 (58.1) | 91.5 (62.5) | .68 | 0.10 |
| IBS-SSS, mean (SD) | 281.1 (79.3) | 254.8 (84.4) | .18 | 0.33 |
| PILL, mean (SD) | 17.7 (8.6) | 19.3 (12.2) | .55 | -0.16 |
| POMS, mean (SD) |  |  |  |  |
| Negative subscale | 14.0 (5.6) | 15.2 (5.6) | .35 | 0.22 |
| Positive subscale | 17.3 (5.2) | 17.2 (5.8) | .96 | 0.01 |
| PSS, mean (SD) | 6.6 (2.7) | 7.1 (2.0) | .32 | 0.21 |

*Note:* BMI = Body mass index; IBS-SE = Irritable Bowel Syndrome Self-Efficacy;

IBS-SSS = IBS Symptom Severity Scale; PILL = Pennebaker Inventory of Limbic

Languidness; POMS = Profile of Mood States; PSS = Perceived Stress Scale.

^a^ Assessed at post-resting state.

**Supplementary Table 2: Baseline characteristics of CBT responders and non-responders in fecal microbiome and metabolomics analyses**

| Variable | Responder  (n = 22) | Non-Responder  (n = 12) | *P*-value | Cohen’s *d* |
| --- | --- | --- | --- | --- |
| Female sex, n (%) | 18 (81.8) | 10 (83.3) | 1.0 |  |
| Age, mean (SD) | 43.7 (13.1) | 48.0 (14.9) | .41 | -0.31 |
| BMI, mean (SD) | 28.7 (5.9) | 27.2 (7.5) | .57 | 0.22 |
| IBS-SE, mean (SD) | 89.9 (25.2) | 97.4 (16.0) | .29 | -0.32 |
| Gracely Scale, mean (SD) |  |  |  |  |
| Unpleasantness of IBS sxs | 10.9 (4.3) | 12.0 (3.4) | .50 | -0.25 |
| Intensity of IBS sxs | 12.9 (4.5) | 14.1 (3.0) | .42 | -0.29 |
| fMRI pain/discomfort | 0.38 (0.50) | 0.56 (0.53) | .41 | -0.34 |
| IBS-SSS, mean (SD) | 288.5 (71.5) | 292.8 (66.2) | .86 | -0.06 |
| PILL, mean (SD) | 17.9 (7.7) | 23.0 (11.4) | .18 | -0.54 |
| PSS, mean (SD) | 6.7 (3.1) | 8.3 (3.2) | .16 | -0.52 |
| POMS, mean (SD) |  |  |  |  |
| Negative subscale | 15.6 (5.8) | 16.4 (6.3) | .75 | -0.13 |
| Positive subscale | 15.4 (6.5) | 17.7 (6.5) | .41 | -0.34 |
| BSS, mean (SD) | 3.8 (1.7) | 4.0 (2.0) | .79 | -0.10 |

*Note:* BMI = Body mass index; IBS-SE = Irritable Bowel Syndrome Self-Efficacy;

IBS-SSS = IBS Symptom Severity Scale; PILL = Pennebaker Inventory of Lambic

Languidness; PSS = Perceived Stress Scale; BSS = Bristol Stool Scale.

**Supplementary Table 3. Differential baseline fecal metabolites in CBT responders vs. non-responders**

| **Metabolite** | **Super Pathway** | **Sub Pathway** | **Log2 Fold Change** | ***P*-value** | ***Q*-value** |
| --- | --- | --- | --- | --- | --- |
| Biochanin A | Xenobiotics | Food Component/Plant | -0.79 | .002 | .64 |
| Beta-cryptoxanthin | Cofactors and Vitamins | Vitamin A Metabolism | 1.11 | .003 | .64 |
| N-stearoyl-sphinganine (d18:0/18:0) | Lipid | Sphingolipid Metabolism | 0.84 | .003 | .64 |
| Delta-tocopherol | Cofactors and Vitamins | Tocopherol Metabolism | 0.89 | .003 | .64 |
| Phenylacetylglycine | Peptide | Acetylated Peptides | -0.80 | .006 | .64 |
| Arabonate/xylonate | Carbohydrate | Pentose Metabolism | -0.64 | .006 | .64 |
| Guanidinoacetate | Amino Acid | Creatine Metabolism | -1.69 | .006 | .64 |
| Creatine | Amino Acid | Creatine Metabolism | -1.28 | .007 | .64 |
| Phytanate | Xenobiotics | Food Component/Plant | 1.26 | .007 | .64 |
| Hexadecanedioate (C16-DC) | Lipid | Fatty Acid, Dicarboxylate | 0.71 | .008 | .64 |
| Myristoleate (14:1n5) | Lipid | Long Chain Fatty Acid | 0.86 | .010 | .64 |
| Serotonin | Amino Acid | Tryptophan Metabolism | 0.88 | .012 | .64 |
| Gamma-tocopherol/beta-tocopherol | Cofactors and Vitamins | Tocopherol Metabolism | 0.63 | .012 | .64 |
| Pristanate | Lipid | Fatty Acid, Branched | 1.06 | .012 | .64 |
| Argininosuccinate | Amino Acid | Urea cycle; Arginine and Proline Metabolism | -0.81 | .014 | .64 |
| 6-hydroxynicotinate | Cofactors and Vitamins | Nicotinate and Nicotinamide Metabolism | -1.05 | .014 | .64 |
| Glutarate (C5-DC) | Lipid | Fatty Acid, Dicarboxylate | 1.31 | .014 | .64 |
| Chrysoeriol | Xenobiotics | Food Component/Plant | -0.57 | .014 | .64 |
| Cadaverine | Amino Acid | Lysine Metabolism | -1.21 | .015 | .64 |
| Apigenin | Xenobiotics | Food Component/Plant | -0.92 | .016 | .64 |
| Palmitoleoylcarnitine (C16:1) | Lipid | Fatty Acid Metabolism(Acyl Carnitine) | 1.11 | .018 | .64 |
| 5-dodecenoate (12:1n7) | Lipid | Medium Chain Fatty Acid | 1.09 | .019 | .64 |
| 5-aminovalerate | Amino Acid | Lysine Metabolism | -0.84 | .020 | .64 |
| Glycyrrhetinate | Xenobiotics | Food Component/Plant | -0.90 | .020 | .64 |
| Ceramide (d18:1/14:0, d16:1/16:0) | Lipid | Ceramides | 0.74 | .020 | .64 |
| Dimethylglycine | Amino Acid | Glycine, Serine and Threonine Metabolism | -0.40 | .021 | .64 |
| Xanthosine | Nucleotide | Purine Metabolism, (Hypo)Xanthine/Inosine containing | 0.95 | .021 | .64 |
| Arginine | Amino Acid | Urea cycle; Arginine and Proline Metabolism | -1.00 | .022 | .64 |
| Mevalonate | Lipid | Mevalonate Metabolism | -1.04 | .022 | .64 |
| Enterolactone | Xenobiotics | Food Component/Plant | 1.46 | .022 | .64 |
| Gabapentin | Xenobiotics | Drug - Neurological | -1.82 | .023 | .64 |
| 1-palmitoyl-2-oleoyl-GPC (16:0/18:1) | Lipid | Phosphatidylcholine (PC) | -0.82 | .023 | .64 |
| Guanosine | Nucleotide | Purine Metabolism, Guanine containing | 1.22 | .024 | .65 |
| 4-hydroxyhippurate | Xenobiotics | Benzoate Metabolism | -0.80 | .028 | .71 |
| Diosmetin | Xenobiotics | Food Component/Plant | -0.41 | .031 | .75 |
| 1-(1-enyl-oleoyl)-GPE (P-18:1) | Lipid | Lysoplasmalogen | -0.66 | .031 | .75 |
| N-stearoyl-sphingosine (d18:1/18:0) | Lipid | Ceramides | 0.87 | .032 | .75 |
| 1-palmitoyl-2-linolenoyl-digalactosylglycerol (16:0/18:3) | Xenobiotics | Food Component/Plant | -0.71 | .034 | .77 |
| Chenodeoxycholate | Lipid | Primary Bile Acid Metabolism | -1.58 | .036 | .77 |
| Ceramide (d18:1/20:0, d16:1/22:0, d20:1/18:0) | Lipid | Ceramides | 1.04 | .037 | .77 |
| 5alpha-pregnan-3beta,20beta-diol monosulfate (1) | Lipid | Progestin Steroids | 1.25 | .039 | .77 |
| Hexadecasphingosine (d16:1) | Lipid | Sphingolipid Metabolism | 0.99 | .041 | .77 |
| 10-heptadecenoate (17:1n7) | Lipid | Long Chain Fatty Acid | 0.67 | .041 | .77 |
| Piperine | Xenobiotics | Food Component/Plant | 1.56 | .042 | .77 |
| 1,2-dipalmitoyl-GPC (16:0/16:0) | Lipid | Phosphatidylcholine (PC) | -0.72 | .042 | .77 |
| Ceramide (d16:1/24:1, d18:1/22:1) | Lipid | Ceramides | 1.10 | .044 | .77 |
| Naringenin | Xenobiotics | Food Component/Plant | -0.86 | .044 | .77 |
| Lanosterol | Lipid | Sterol | 0.61 | .045 | .77 |
| Pyridoxine (Vitamin B6) | Cofactors and Vitamins | Vitamin B6 Metabolism | -1.04 | .046 | .77 |
| 1-linoleoyl-2-linolenoyl-galactosylglycerol (18:2/18:3) | Lipid | Glycolipid Metabolism | -0.77 | .047 | .77 |
| 1-palmitoyl-2-oleoyl-GPE (16:0/18:1) | Lipid | Phosphatidylethanolamine (PE) | -1.00 | .047 | .77 |
| Pseudoephedrine | Xenobiotics | Drug - Respiratory | -0.87 | .048 | .77 |
| M-tyramine | Amino Acid | Tyrosine Metabolism | 0.71 | .049 | .77 |
| 1-(1-enyl-stearoyl)-GPE (P-18:0) | Lipid | Lysoplasmalogen | -0.72 | .049 | .77 |
| Theophylline | Xenobiotics | Xanthine Metabolism | 0.54 | .050 | .77 |

**Supplementary Table 4: Baseline macronutrient and tryptophan intake in CBT responders and non-responders**

|  | Responder  (n = 22) | Non-Responder  (n = 12) | *P*-value | Cohen’s *d* |
| --- | --- | --- | --- | --- |
| Energy Intake (kcal/day) | 1912 (834) | 1545 (1006) | .13 | 0.40 |
| % Energy from Carbohydrates | 45.1 (8.0) | 52.8 (14.1) | **.03** | **-0.72** |
| % Energy from Protein | 15.9 (1.8) | 15.2 (3.4) | .82 | 0.29 |
| % Energy from Fat | 39.2 (9.2) | 32.1 (9.1) | **.04** | **0.75** |
| % Energy from Saturated Fat | 12.0 (2.4) | 10.1 (3.1) | *.09* | *0.69* |
| % Energy from Monounsaturated Fat | 15.2 (4.9) | 11.7 (3.7) | **.03** | **0.75** |
| % Energy from Polyunsaturated Fat | 8.5 (3.2) | 7.1 (2.3) | .35 | 0.43 |
| % Energy from Alcohol | 2.4 (4.3) | 2.1 (3.5) | .70 | 0.07 |
| Dietary Fiber (g/day) | 17.3 (9.6) | 14.5 (11.9) | *.09* | *0.26* |
| Tryptophan (g/day) | 0.87 (0.40) | 0.66 (0.54) | .21 | 0.47 |

Note: Values expressed as mean (SD)

**Supplementary Table 5: Post-CBT changes in clinical measures in CBT responders and non-responders who underwent neuroimaging**

|  | Responders (n = 58) | | | |
| --- | --- | --- | --- | --- |
| Variable | Pre | Post | *P*-value | Cohen’s *d* |
| Gracely Scale |  |  |  |  |
| Unpleasantness of IBS sxs | 10.7 (4.1) | 6.7 (5.2) | **< .001** | **-0.86** |
| Intensity of IBS sxs | 12.2 (3.9) | 6.8 (5.0) | **< .001** | **-1.20** |
| fMRI pain/discomfort ^a^ | 0.32 (0.47) | 0.26 (0.44) | .520 | -0.14 |
| IBS-SE | 97.6 (58.1) | 191.6 (61.6) | **< .001** | **1.57** |
| IBS-SSS | 281.1 (79.3) | 152.6 (83.5) | **< .001** | **-1.58** |
| PILL | 17.7 (8.6) | 19.3 (9.7) | **.005** | **0.25** |
| POMS |  |  |  |  |
| Negative subscale | 14.0 (5.6) | 11.5 (7.0) | **.002** | **-0.39** |
| Positive subscale | 17.3 (5.2) | 20.3 (5.7) | **< .001** | **0.55** |
| PSS | 6.6 (2.7) | 5.0 (2.9) | **< .001** | **-0.56** |

|  | Non-Responders (n = 26) | | | |
| --- | --- | --- | --- | --- |
| Variable | Pre | Post | *P*-value | Cohen’s *d* |
| Gracely Scale |  |  |  |  |
| Unpleasantness of IBS sxs | 10.3 (3.2) | 9.3 (5.4) | .42 | -0.22 |
| Intensity of IBS sxs | 12.0 (4.5) | 9.6 (5.3) | **.03** | **-0.49** |
| fMRI pain/discomfort ^a^ | 0.38 (0.50) | 0.35 (0.49) | .66 | -0.08 |
| IBS-SE | 91.5 (62.5) | 138.5 (61.4) | **.001** | **0.76** |
| IBS-SSS | 254.8 (84.4) | 261.0 (93.5) | .52 | 0.07 |
| PILL | 19.3 (12.2) | 24.7 (11.0) | **< .001** | **0.47** |
| POMS |  |  |  |  |
| Negative subscale | 15.2 (5.6) | 13.4 (6.3) | *.09* | *-0.31* |
| Positive subscale | 17.2 (5.8) | 16.7 (6.7) | .87 | -0.07 |
| PSS | 7.1 (2.0) | 6.4 (3.5) | .24 | -0.24 |

*Note:* IBS-SE = Irritable Bowel Syndrome Self-Efficacy; IBS-SSS = IBS Symptom Severity Scale; PILL = Pennebaker Inventory of Lambic Languidness; POMS = Profile of Mood States; PSS = Perceived Stress Scale.

^a^ Assessed at post resting state.

**Supplementary Table 6: Post-CBT changes in clinical measures in CBT responders and non-responders in fecal microbiome and metabolomics analyses**

|  | Responders (n = 22) | | | |
| --- | --- | --- | --- | --- |
| Variable | Pre | Post | *P*-value | Cohen’s *d* |
| Gracely Scale |  |  |  |  |
| Unpleasantness of IBS sxs | 10.9 (4.3) | 6.0 (5.9) | **.02** | **-0.66** |
| Intensity of IBS sxs | 12.9 (4.5) | 4.7 (4.6) | **< .001** | **-1.45** |
| fMRI pain/discomfort ^a^ | 0.38 (0.50) | 0.25 (0.45) | .33 | -0.25 |
| IBS-SE | 89.9 (25.2) | 165.0 (73.1) | **.002** | **0.96** |
| IBS-SSS | 288.5 (71.5) | 145.1 (89.4) | **< .001** | **-1.85** |
| PILL | 17.9 (7.7) | 17.3 (9.0) | .81 | -0.06 |
| POMS |  |  |  |  |
| Negative subscale | 15.4 (6.5) | 11.8 (6.6) | **.02** | **-0.65** |
| Positive subscale | 15.6 (5.8) | 19.6 (7.1) | **.01** | **0.73** |
| PSS | 6.7 (3.1) | 5.7 (2.7) | **.02** | **-0.65** |

|  | Non-Responders (n = 12) | | | |
| --- | --- | --- | --- | --- |
| Variable | Pre | Post | *P*-value | Cohen’s *d* |
| Gracely Scale |  |  |  |  |
| Unpleasantness of IBS sxs | 12.0 (3.4) | 11.2 (5.4) | .74 | -0.11 |
| Intensity of IBS sxs | 14.1 (3.0) | 12.4 (5.3) | .36 | -0.33 |
| fMRI pain/discomfort ^a^ | 0.56 (0.53) | 0.56 (0.53) | 1.0 | 0.00 |
| IBS-SE | 97.4 (16.0) | 137.8 (78.5) | **.04** | **0.82** |
| IBS-SSS | 292.8 (66.2) | 296.8 (62.7) | .69 | 0.12 |
| PILL | 23.0 (11.4) | 30.1 (11.6) | **.01** | **1.04** |
| POMS |  |  |  |  |
| Negative subscale | 17.7 (6.5) | 16.8 (9.6) | .61 | -0.18 |
| Positive subscale | 16.4 (6.3) | 15.3 (8.6) | .19 | -0.17 |
| PSS | 8.3 (3.2) | 8.0 (3.5) | .87 | -0.06 |

*Note:* IBS-SE = Irritable Bowel Syndrome Self-Efficacy; IBS-SSS = IBS Symptom Severity Scale; PILL = Pennebaker Inventory of Lambic Languidness; POMS = Profile of Mood States; PSS = Perceived Stress Scale.

^a^ Assessed at post resting state.

**Supplementary Table 7. Differential metabolites in CBT responders at the end of treatment compared to baseline**

| **Metabolite** | **Super Pathway** | **Sub Pathway** | **Log2 Fold Change** | ***P*-value** | ***Q*-value** |
| --- | --- | --- | --- | --- | --- |
| Arabitol/xylitol | Carbohydrate | Pentose Metabolism | 0.76 | .002 | .94 |
| 2-isopropylmalate | Xenobiotics | Food Component/Plant | 0.62 | .002 | .94 |
| 3-hydroxy-3-methylglutarate | Lipid | Mevalonate Metabolism | 1.05 | .004 | .98 |
| Glucuronate | Carbohydrate | Aminosugar Metabolism | 0.44 | .005 | .98 |
| Erucoylcarnitine (C22:1) | Lipid | Fatty Acid Metabolism(Acyl Carnitine) | -0.57 | .008 | .98 |
| Cysteinylglycine | Amino Acid | Glutathione Metabolism | 0.51 | .009 | .98 |
| Diosmetin | Xenobiotics | Food Component/Plant | 0.79 | .009 | .98 |
| Inosine | Nucleotide | Purine Metabolism, (Hypo)Xanthine/Inosine containing | -0.43 | .011 | .98 |
| Glycerophosphoserine | Lipid | Phospholipid Metabolism | 0.55 | .012 | .98 |
| Glycerophosphoethanolamine | Lipid | Phospholipid Metabolism | 0.34 | .013 | .98 |
| Guanosine | Nucleotide | Purine Metabolism, Guanine containing | -0.57 | .014 | .98 |
| Enterolactone | Xenobiotics | Food Component/Plant | -0.51 | .018 | .98 |
| 1,2-dilinolenoyl-galactosylglycerol (18:3/18:3) | Lipid | Glycolipid Metabolism | 1.13 | .020 | .98 |
| Arachidoylcarnitine (C20) | Lipid | Fatty Acid Metabolism(Acyl Carnitine) | -0.49 | .021 | .98 |
| 1-palmitoyl-2-linolenoyl-digalactosylglycerol (16:0/18:3) | Xenobiotics | Food Component/Plant | 0.68 | .021 | .98 |
| Liquiritigenin | Xenobiotics | Food Component/Plant | 0.96 | .022 | .98 |
| Ribonate | Carbohydrate | Pentose Metabolism | 0.47 | .023 | .98 |
| Succinylcarnitine (C4-DC) | Energy | TCA Cycle | 0.53 | .027 | .98 |
| OAHSA (18:1/OH-18:0) | Lipid | Fatty Acid Hydroxyl Fatty Acid | 0.51 | .030 | .98 |
| Behenoylcarnitine (C22) | Lipid | Fatty Acid Metabolism(Acyl Carnitine) | -0.46 | .034 | .98 |
| Gamma-glutamylglycine | Peptide | Gamma-glutamyl Amino Acid | 0.51 | .035 | .98 |
| 1-stearoyl-GPE (18:0) | Lipid | Lysophospholipid | 0.39 | .036 | .98 |
| Vanillactate | Amino Acid | Tyrosine Metabolism | 0.36 | .039 | .98 |
| N-methylglutamate | Amino Acid | Glutamate Metabolism | 0.32 | .040 | .98 |
| N-acetylmethionine sulfoxide | Amino Acid | Methionine, Cysteine, SAM and Taurine Metabolism | 0.33 | .040 | .98 |
| 5-aminovalerate | Amino Acid | Lysine Metabolism | 0.32 | .040 | .98 |
| Hexadecatrienoate (16:3n3) | Lipid | Polyunsaturated Fatty Acid (n3 and n6) | 0.70 | .040 | .98 |
| LAHSA (18:2/OH-18:0) | Lipid | Fatty Acid Hydroxyl Fatty Acid | 0.44 | .041 | .98 |
| Phenylacetate | Amino Acid | Phenylalanine Metabolism | -0.46 | .041 | .98 |
| Fucose | Carbohydrate | Pentose Metabolism | 0.28 | .044 | .98 |
| PAHSA (16:0/OH-18:0) | Lipid | Fatty Acid Hydroxyl Fatty Acid | 0.50 | .044 | .98 |
| Indole | Amino Acid | Tryptophan Metabolism | -0.51 | .047 | .98 |
| 1-stearoyl-GPS (18:0) | Lipid | Lysophospholipid | 0.67 | .047 | .98 |
| 2'-deoxyinosine | Nucleotide | Purine Metabolism, (Hypo)Xanthine/Inosine containing | -0.44 | .047 | .98 |
| Phosphate | Energy | Oxidative Phosphorylation | 0.61 | .048 | .98 |
| 1-(1-enyl-palmitoyl)-GPE (P-16:0) | Lipid | Lysoplasmalogen | 0.29 | .049 | .98 |
| Solanidine | Xenobiotics | Food Component/Plant | -0.98 | .050 | .98 |

**Supplementary Table 8: Macronutrient and tryptophan intake at baseline and after CBT in responders and non-responders**

|  | Responders (n = 22) | | | |
| --- | --- | --- | --- | --- |
|  | Pre | Post | *P*-value | Cohen’s *d* |
| Energy Intake (kcal/day) | 1912 (834) | 1857 (773) | .24 | -0.07 |
| % Energy from Carbohydrates | 45.1 (8.0) | 46.2 (8.2) | .78 | 0.13 |
| % Energy from Protein | 15.9 (1.8) | 16.1 (1.9) | .29 | 0.09 |
| % Energy from Fat | 39.2 (9.2) | 38.6 (8.4) | .99 | -0.06 |
| % Energy from Saturated Fat | 12.0 (2.4) | 11.1 (1.5) | .13 | -0.46 |
| % Energy from Monounsaturated Fat | 15.2 (4.9) | 15.2 (4.6) | .68 | -0.003 |
| % Energy from Polyunsaturated Fat | 8.5 (3.2) | 8.8 (3.0) | .32 | 0.12 |
| % Energy from Alcohol | 2.4 (4.3) | 1.9 (2.5) | .91 | -0.13 |
| Dietary Fiber (g/day) | 17.3 (9.6) | 18.5 (10.9) | .92 | 0.12 |
| Tryptophan (g/day) | 0.87 (0.40) | 0.87 (0.37) | .98 | -0.003 |

|  | Non-Responders (n = 12) | | | |
| --- | --- | --- | --- | --- |
|  | Pre | Post | *P*-value | Cohen’s *d* |
| Energy Intake (kcal/day) | 1545 (1006) | 1565 (970) | .97 | 0.02 |
| % Energy from Carbohydrates | 38.2 (14.1) | 55.4 (14.1) | .57 | 0.18 |
| % Energy from Protein | 15.2 (3.4) | 14.1 (3.8) | .23 | -0.29 |
| % Energy from Fat | 32.1 (9.1) | 31.6 (10.5) | .79 | -0.06 |
| % Energy from Saturated Fat | 10.1 (3.1) | 9.1 (3.2) | **.03** | **-0.32** |
| % Energy from Monounsaturated Fat | 11.7 (3.7) | 11.8 (3.9) | .91 | 0.03 |
| % Energy from Polyunsaturated Fat | 7.1 (2.3) | 7.5 (3.3) | .52 | -0.14 |
| % Energy from Alcohol | 2.1 (3.5) | 1.6 (2.4) | .54 | -0.16 |
| Dietary Fiber (g/day) | 14.5 (11.9) | 16.1 (15.1) | .73 | 0.12 |
| Tryptophan (g/day) | 0.66 (0.54) | 0.63 (0.49) | .87 | -0.06 |

Note: Values expressed as mean (SD)
